# Enhancing Near-Infrared Absorption in Terpyridyl Ru/Os Complexes with Ancillary Ligands to Activate Spin-Forbidden Transitions in Dye-Sensitized Solar Cells: A TDDFT Investigation

**DOI:** 10.1021/acs.jpca.3c07554

**Published:** 2024-01-25

**Authors:** Ratna Juwita, Jian-Ming Liao, Chia-Yuan Chen, Hui-Hsu Gavin Tsai

**Affiliations:** † Applied Science, Universitas Negeri Malang, 551312 Malang, Indonesia; ‡ Department of Chemistry, National Central University, No. 300, Zhongda Road, Zhongli District, Taoyuan City 32001, Taiwan; § Research Center of New Generation Light Driven Photovoltaic Module, National Central University, No. 300, Zhongda Road, Zhongli District, Taoyuan City 32001, Taiwan

## Abstract

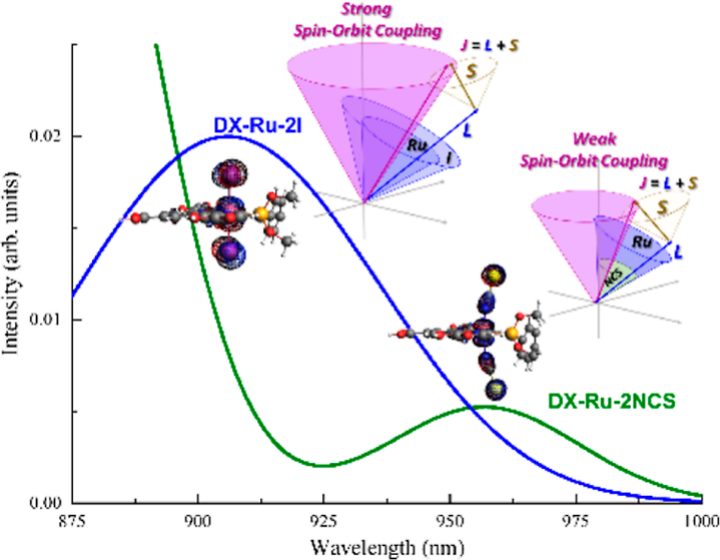

Dye sensitizers with wideband absorption covering the near-IR region have long been of interest because they potentially harvest a wide range of solar energies essential to promote photocurrent power conversion efficiencies. In this study, we used time-dependent density functional theory with spin–orbit (SO) interactions to theoretically explore the long-wavelength absorptions and spin-forbidden triplet transitions activated by SO interactions for terpyridyl ruthenium/osmium complex dyes. These dyes feature a Ru(II) sensitizer coordinated with a phosphine ligand and are exemplified by DX1, denoted as [*trans*-dichloro-(phenyldimethoxyphosphine)(2,2′;6′,2″-terpyridyl-4,4′,4″-tricarboxylic)Ru]. We found that ancillary ligands significantly affected the longest wavelength spin-allowed absorption, with NCS^–^ ligands yielding longer wavelength S_1_ transitions than halides. High atomic number halide ligands caused blue shifts in the S_1_ transition. Os complexes consistently exhibited longer wavelength S_1_ transitions than Ru complexes with identical ligands. In Ru/Os complexes, ancillary ligands with higher atomic numbers have a more pronounced effect in activating spin-forbidden triplet transitions through spin–orbit coupling (SOC) than those with lower atomic numbers. The absorption wavelength of the SOC-activated transition primarily depended on the energy of lower lying triplet states. Some complexes exhibited T_1_ states activated by SOC, leading to longer wavelength absorption than that of SOC-activated T_2_ states. Our study revealed the significance of ancillary ligands and SOC interactions in Ru/Os complexes, offering insights for optimizing materials with enhanced long-wavelength absorption properties, particularly in the near-IR range, for photovoltaic and optoelectronic applications.

## Introduction

Dye-sensitized solar cells (DSSCs) are promising and cost-effective devices for indoor and building-integrated photovoltaic (BIPV) applications.^1−3^ The essential components of DSSCs encompass a porous semiconductor thin film, dye sensitizers, a counter electrode, and a redox couple, in which the dye sensitizers play a pivotal role in light absorption and charge separation. To enhance the alignment between solar radiation and the dye absorption spectrum, design principles are employed to broaden the sensitizers’ absorption to longer wavelengths, including the near-infrared (NIR) region.^2−5^ This approach not only achieves a panchromatic response in solar cells but also enhances the feasibility of tandem and light-splitting device configurations. Various strategies have been explored to extend the absorption of dyes into the NIR region. These strategies include extending the conjugation length,^2,3,6−9^ introducing functional moieties^10−13^ such as squaraine, and enhancing the spin–orbit (SO) interactions^14−24^ of sensitizers. The first two methods aim to narrow the disparity between the highest occupied molecular orbital (HOMO) and the lowest unoccupied molecular orbital (LUMO) of dye sensitizers. Nevertheless, organic compounds with reduced HOMO–LUMO gaps often face challenges in achieving efficient photoelectric conversion due to inherent losses during photoinduced charge separation. Furthermore, altering a dye to shift its absorption toward longer wavelengths may not necessarily result in an overall increase in absorption efficiency.

SO interactions have been demonstrated to effectively activate longer wavelength absorption from spin-forbidden triplet states, leading to a broader absorption range for sensitizers and enhancing the spectral response of the devices.^18−22,25^ The SO interaction plays a crucial role in mixing both spin-allowed singlet and spin-forbidden triplet excitations, which can be induced by the electron spin–orbit coupling (SOC) within the electric field of the nuclei.^21,26^ The oscillator strength of spin-forbidden triplet transitions borrows intensity from spin-allowed singlet transitions.^26^ Enhanced SOC can activate the low-lying, spin-forbidden triplet state, resulting in longer wavelength absorption compared to that of the corresponding spin-allowed singlet states. This, in turn, enhances the photovoltaic performance of the devices.^16,20,27,28^ The oscillator strength of a spin-forbidden triplet excited state is influenced by both the energy gap between the singlet and triplet excited states and their respective SOC constants when activated by SOC.^16^ Designing dyes with enhanced NIR absorptivity for DSSC applications offer several advantages. SOC effects activate the spin-forbidden triplet states of sensitizers, providing additional longer wavelength absorptions and reducing recombination between singlet excited and ground states through direct transitions to the triplet excited state.^23^ Furthermore, the longer lifetime of the triplet state, compared with that of the singlet state, favors electron injection from the sensitizer to the semiconductor. A recent variant of black dye, known as DX1, exhibits enhanced spectral intensity in the 700–900 nm range, attributed to spin-forbidden singlet–triplet transitions.^18^ A tandem-type DSSC incorporating both DX1 and the conventional sensitizer N719 demonstrates a power conversion efficiency exceeding 12%^18^ when subjected to simulated sunlight with an intensity of 35.5 mW/cm^2^.

Ru(II) sensitizers in DSSCs have been reported to efficiently convert light from the visible to NIR regions by utilizing spin-forbidden transitions.^18,19,25^ DSSCs employing the black dye,^29^ [Ru(4,4′,4′–COOH–2,2′;6,2′-tpy)(NCS)_3_], have demonstrated optical absorption extending up to 900 nm and achieved a certified energy conversion efficiency of 11.9%. *trans*-[RuCl_2_(phenyldimethoxyphosphine)-(4,4′,4′–COOH–2,20;6′,2′-tpy)] (DX1)^18^ is a highly efficient Ru complex that exhibits a long-wavelength absorption that arises from triplet metal-to-ligand charge transfer (^3^MLCT) activated through SO interactions. DX1 has the same 4,4′,4′–COOH–2,2′;6,2′-tpy anchoring ligand as BD and three ancillary ligands (one phenyldimethoxyphosphine unit and two chloride anions). Although strong SO interactions are common in molecules containing heavy atoms, long-wavelength absorptions activated by SO interactions have not been reported for most Ru dyes. Moreover, a DX1-based Fe complex has exhibited weak SO interactions.^23^

A high atomic number atom within a molecule enhances spin–orbit coupling (SOC) between singlet and triplet states, resulting in more effective intersystem crossing (ISC).^30^ Several osmium(II) (Os) complexes have exhibited suitable excited- and ground-state energy levels, good thermal and chemical stabilities,^24^ and shorter lifetimes of excited ^3^MLCT when compared with corresponding Ru complexes, presumably because the larger SOC constant in Os complexes enhances the rate of ISC from the triplet excited state to the ground state.^31^ The SOC in Os complexes results in an “additional” absorption at longer wavelength that can enhance the solar photon absorption efficiency.^32^ In 2014, Fantacci and colleagues replaced the Ru center in [Ru(bpy)_3_]^2+^ by an Os atom to obtain [Os(bpy)_3_]^2+^, which displayed a low-energy absorption band^21^ and excellent optical properties, with expanded absorption into the NIR region.^17^ Os complexes featuring SOC are promising materials for extending the photocurrent responses of DSSCs. Recently, a new Os complex, CYC-33O, displaying red-shifted absorption and enhanced absorbance of its singlet metal-to-ligand charge transfer (^1^MLCT) and ^3^MLCT transitions,^18^ was demonstrated to give an improved photocurrent response in the NIR region relative to that of its Ru counterpart. In principle, because the heavier Os(II) cation has a larger SOC constant than that of the Ru atom,^15−21^ Os complexes can potentially induce an absorption of the ^3^MLCT.^17^ Therefore, we wished to explore the nature of SO interactions, potentially influencing the strength of SOC in Ru/Os complexes and activating the longer wavelength of absorption through SOC, with the goal of designing high-performance Ru/Os complex photosensitizers.

**Figure 1:**
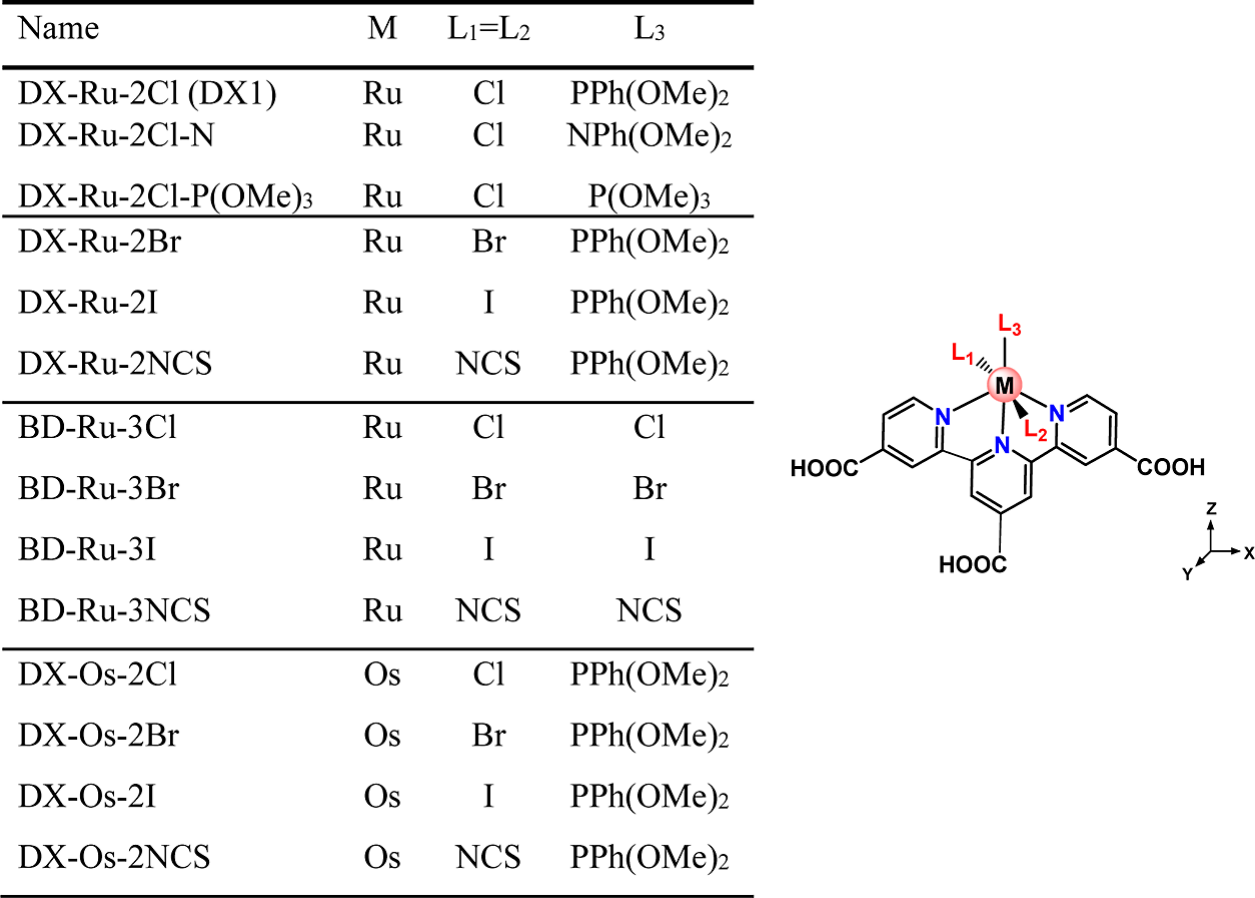
Chemical structures and names of the studied Ru and the Os complex dyes.

The Ru/Os sensitizers exhibit an enhanced ^1^MLCT state, where the electron density is primarily transferred from the metal to the ligand. Since ancillary ligands are coordinated to the metal, this transition also involves the electron density of the ancillary ligands.^20,25^ In this regard, the nature of ancillary ligands may not only influence the absorption of ^1^MLCT but also affect the absorption of ^3^MLCT through SOC. In this study, we conducted a theoretical exploration of the absorption spectra and the characteristics of SOC for 14 dyes based on DX1 and BD-like Ru/Os complexes^23^ (Figure 1). We employed time-dependent density functional theory (TDDFT) with spin–orbit (SO) interactions (SO-TDDFT). Our investigation focused on understanding the impact of different ancillary ligands (Cl^–^, Br^–^, I^–^, NCS^–^, and phenyldimethoxyphosphine-derived ligands) on the long-wavelength spin-forbidden absorptions of these dyes. We aimed to elucidate the nature of SOC effects in Ru/Os complexes and how the ancillary ligands induce these SOC interactions. Additionally, we examined how the presence of heavy Os atoms contributes to the strong SOC effects.

## Computational Methods

All calculations were performed using the Amsterdam density functional program (ADF2017).^33^ The ground-state molecular geometries of all of the studied complexes (Figure 1) were optimized in the gas phase by employing the Becke–Perdew (BP) exchange–correlation functional^21,22^ with zeroth-order regular approximation^34,35^ (ZORA) triple-ζ + polarization (TZP) for Ru, Os, and I atoms (involving core potentials for the cores 1s–3d for Ru and 1s–4d for Os; the cores 1s–4p for I were kept frozen), while the ZORA double-ζ + polarization (DZP) basis sets was used for all other atoms (the cores 1s for C, N, and O; the cores 1s–2p for Cl, S, and P; and the cores 1s–3p for Br). The optimized structures were examined by vibration frequency calculations with all positive values.

Electronic transition energies were calculated through TDDFT including the perturbative SOC approach^36^ with a relativistic Hamiltonian based on the ZORA^34,35^ using the Becke three-parameter Lee–Yang–Parr (B3LYP)^37,38^ functional. The solvent effects of *N*,*N*-dimethylformamide (DMF) were considered by using the conductor-like screening model (COSMO). The effects of scalar relativistic (SR)^39^ and SO coupling on the absorption properties were examined in a self-consistent manner. The optical properties were determined by calculating the singlet–singlet and singlet–triplet transitions of the complexes. Excitation energies were calculated for the 20 excited states in each complex.

## Results and Discussion

A comparative study was conducted to examine the influence of ancillary ligands on the activation of NIR absorption in Ru/Os complexes. This study involved three series of Ru complexes and one series of Os complexes:

(1) The first series of complexes was designed based on the DX-Ru-2Cl (DX1) framework, consisting of three complexes: DX-Ru-2Cl, DX-Ru-2Cl–N, and DX-Ru-2Cl–P(OMe)_3_. This series aimed to investigate the effects of bulky ligands containing P and N.
(2) The second series of complexes was also based on the DX-Ru-2Cl (DX1) framework, comprising three complexes: DX-Ru-2Br, DX-Ru-2I, and DX-Ru-2NCS. These complexes were used to study the impact of halide and commonly used NCS^–^ ligands.
(3) The third series of complexes replaced the PPh(OMe)_2_ ligand of DX-Ru-2Cl with halide or NCS^–^ ligands, resulting in three complexes ligated by the same halide or NCS ligands: DX-Ru-3Cl, DX-Ru-3Br, DX-Ru-3I, and DX-Ru-3NCS. This series aimed to investigate the effects of halide and commonly used NCS^–^ ligands.
(4) The fourth series of complexes consisted of Os complexes designed based on the DX-Ru-2Cl (DX1) framework, including four complexes: DX-Os-2Cl, DX-Os-2Br, DX-Ru-2I, and DX-Ru-2NCS. This series explored the influence of the Os atom as well as ancillary halide and NCS^–^ ligands.

### Effects of Phenyldimethoxyphosphine-Derived Ligands on the Absorption Spectra of DX-Ru-2Cl, DX-Ru-2Cl–N, and DX-Ru-2Cl–P(OMe)_3_

Initially, our investigation focused on scrutinizing the impact of phenyldimethoxyphosphine-derived ligands on the absorption spectra of DX1-based Ru complexes, with particular emphasis on the SOC effects induced by these ligands. Table 1 provides an overview of the characteristics of six frontier molecular orbitals (H – 2, H – 1, H, L, L + 1, and L + 2) for DX-Ru-2Cl (the original DX1), DX-Ru-2Cl–N, and DX-Ru-2Cl–P(OMe)_3_. The MOs and their associated energy levels are illustrated in Figure S1 (Supporting Information). Our analysis revealed that the Ru 4d orbitals dominate the three highest occupied MOs (H – 2, H – 1, and H). The Cl^–^ ligands also made contributions to the two highest occupied MOs. In contrast, the ancillary ligands, namely, phenyldimethoxyphosphine (*pdp*), phenyldimethoxynitryl (*pdn*), or trimethoxyphosphine (*tmp*), exhibited minimal contributions to the orbital properties of the six frontier MOs. Conversely, the three lowest unoccupied MOs (L, L + 1, and L + 2) were primarily governed by the π* orbitals of the anchoring ligand tricarboxyterpyridine (*tctpy*, 2,2′;6′,2′-terpyridyl-4,4′,4′-tricarboxylic acid). Notably, the DX-Ru-2Cl, DX-Ru-2Cl–N, and DX-Ru-2Cl–P(OMe)_3_ complexes exhibited comparable H–L energy gaps (HLGs). Consequently, these complexes displayed similar S_1_ transitions, primarily originating from HOMO to LUMO transitions, within the wavelength range of 813–822 nm (Table 2).

**Table 1:** Characteristics of Frontier Orbitals of DX-Ru-2Cl, DX-Ru-2Cl–N, and DX-Ru-2Cl–P(OMe)_3_^a^^,^^b^

molecules	orbital	*E* (eV)	orbital character (%) of Ru	orbital character (%) of Cl	orbital character (%) of *pdp/pdn/tmp*	orbital character (%) of *tctpy*
DX-Ru-2Cl (DX1)	L + 2	–2.591	0	0	0	93
	L + 1	–2.884	6d_*xy*_	0	0	88
	L	–3.345	8d_*yz*_	0	0	82
	H	–5.731	56d_*xy*_	24p_*x*_	0	11
	H – 1	–5.867	48d_*yzy*_	30p_*z*_	0	9
	H – 2	–6.503	66d_*xz*_, 6d_*z*_^2^	5p_*x*_	4	4
DX-Ru-2Cl–N	L + 2	–2.565	2d_*yz*_	0	12	79
	L + 1	–2.837	6d_*xy*_	0	8	88
	L	–3.248	12d_*yz*_	0	18	61
	H	–5.708	56d_*xy*_	25p_*x*_	0	11
	H – 1	–5.780	44d_*yz*_	25p_*z*_	0	13
	H – 2	–6.305	70d_*xz*_, 6d_*z*_^2^	0	0	0
DX-Ru-2Cl–P (OMe)_3_	L + 2	–2.607	0	0	0	94
	L + 1	–2.905	6d_*xy*_	0	0	88
	L	–3.386	7d_*yz*_	0	0	84
	H	–5.771	57d_*xy*_	25p_*x*_	0	11
	H – 1	–5.938	50d_*yz*_	31p_*z*_	0	8
	H – 2	–6.620	70d_*xz*_, 5d_*z*_^2^	6p_*x*_	5	0

a *pdp* = [PPh(OMe)_2_] = phenyldimethoxyphosphine, *pdn* = [NPh(OMe)_2_] = phenyldimethoxynitryl, *tmp* = [P(OMe)_3_] = trimethoxyphosphine, and *tctpy* = tricarboxyterpyridine.

b Orbital density below 5% is not listed.

**Table 2:** Calculated SOC-Induced Long-Wavelength Transitions of DX-Ru-2Cl, DX-Ru-2Cl–N, and DX-Ru-2Cl–P(OMe)_3_ and Their Characteristics Contributed in Terms of SR Transitions^a^

perturbative SOC transition	transition assignments	SR contribution	SR composition
DX-Ru-2Cl			
ST_4_: 1.37 eV (907 nm), *f* = 0.007	^3^MLCT	88% T_2_: 1.39 eV (894 nm), *f* = 0.000	90% (H – 1 → L) 5% (H → L)
		10% S_1_: 1.51 eV (822 nm), *f* = 0.073	97% (H → L)
ST_7_: 1.52 eV (814 nm), *f* = 0.065	^1^MLCT	87% S_1_: 1.51 eV (822 nm), *f* = 0.073	97% (H → L)
		10% T_2_: 1.39 eV (894 nm), *f* = 0.000	90% (H – 1 → L) 5% (H → L)
DX-Ru-2Cl–N			
ST_3_: 1.20 eV (1036 nm), *f* = 0.002	^3^MLCT	96% T_1_: 1.21 eV (1024 nm), *f* = 0.000	91% (H – 1 → L)
		3% S_1_: 1.52 eV (816 nm), *f* = 0.059	96% (H → L)
ST_7_: 1.52 eV (816 nm), *f* = 0.056	^1^MLCT	95% S_1_: 1.52 eV (816 nm), *f* = 0.059	96% (H → L)
		3% T_1_: 1.21 eV (1024 nm), *f* = 0.000	91% (H – 1 → L)
DX-Ru-2Cl–P(OMe)_3_			
ST_4_: 1.43 eV (866 nm), *f* = 0.017	^3^MLCT	77% T_2_: 1.46 eV (848 nm), *f* = 0.000	92% (H – 1 → L) 3% (H → L)
		22% S_1_: 1.53 eV (813 nm), *f* = 0.076	98% (H → L)
ST_7_: 1.55 eV (801 nm), *f* = 0.059	^1^MLCT	77% S_1_: 1.53 eV (813 nm), *f* = 0.076	98% (H → L)
		22% T_2_: 1.46 eV (848 nm), *f* = 0.000	92% (H – 1 → L) 3% (H → L)

a SR compositions by MOs. Transitions with an oscillator strength lower than 0.001 are not shown here.

**Figure 2:**
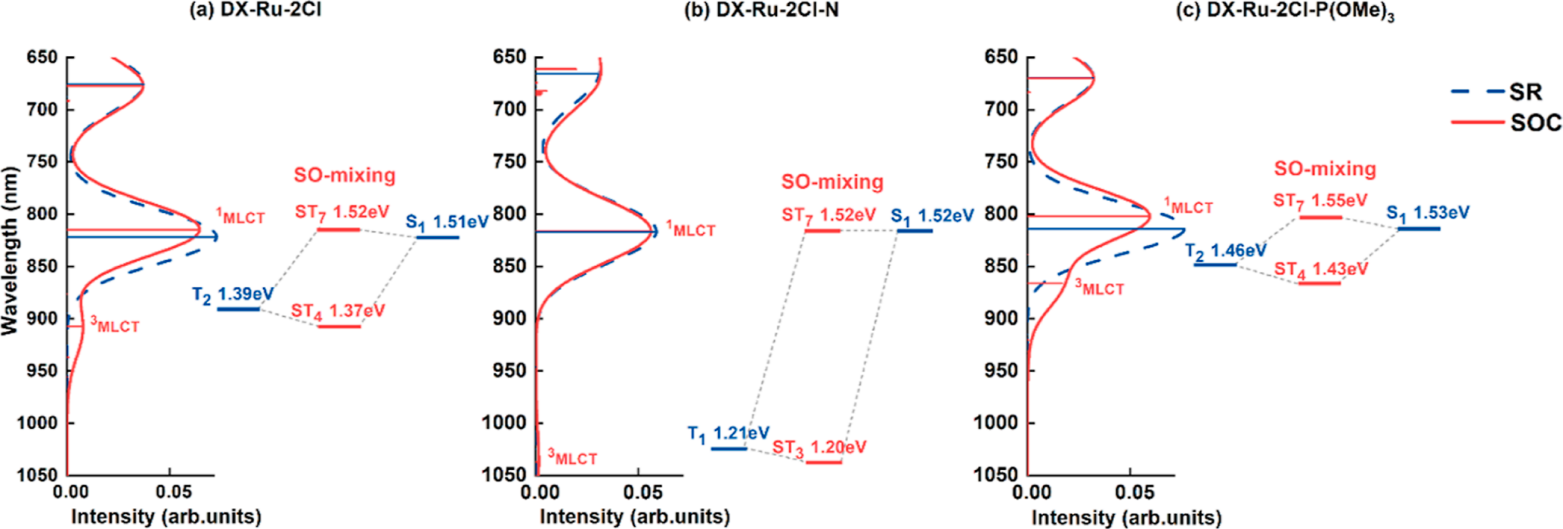
Simulated absorption spectra of (a) DX-Ru-2Cl, (b) DX-Ru-2Cl–N, and (c) DX-Ru-2Cl–P(OMe)_3_ complexes in DMF. Excitation energies and spectra calculated by SR-TDDFT are shown in blue, and excitation energies and spectra calculated by SO-TDDFT are shown in red. An energy diagram illustrating the SO mixing of singlet and triplet excited states, as calculated by SR-TDDFT, is presented alongside the spectra. The ST state represents the SO-mixed state of singlet and triplet excited states.

Figure 2 presents the simulated absorption spectra of DX-Ru-2Cl, DX-Ru-2Cl–N, and DX-Ru-2Cl–P(OMe)_3_, specifically focusing on absorption wavelengths exceeding 650 nm, as calculated employing both spin-restricted time-dependent density functional theory (SR-TDDFT) and spin–orbit time-dependent density functional theory (SO-TDDFT). The simulated absorption spectra for wavelengths exceeding 400 nm are provided in Figure S2 for reference. Table 2 details the salient SOC-induced long-wavelength transitions observed in DX-Ru-2Cl, DX-Ru-2Cl–N, and DX-Ru-2Cl–P(OMe)_3_, along with their relevant characteristics with respect to the SR transitions. For a comprehensive compilation of the calculated transitions for these molecules, please refer to Tables S1–S3 in the Supporting Information. The observed SOC effects primarily activate low-lying spin-forbidden triplet transitions, resulting in nonzero oscillator strengths and red-shifted absorptions. In contrast, the higher lying absorption peaks exhibit minimal shifts that can be attributed to the SO interactions.

The mixture between spin-allowed singlet and spin-forbidden triplet states can be explained by considering the spin-forbidden transition oscillator strength (*f*_ST_) based on perturbation theory^40^
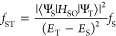
1where Ψ_S_ and Ψ_T_ are the wave functions of the singlet and triplet excited states, respectively; *H*_SO_ is the SOC Hamiltonian; *E*_S_ and *E*_T_ are energies of the singlet and triplet excited states, respectively; and *f*_S_ is the oscillator strength of the spin-allowed singlet transition. In the case of DX-Ru-2Cl, the SO interactions between the S_1_ and spin-forbidden T_2_ states, as depicted in Figure 2a, gave rise to the lowest lying ST_4_ state at 907 nm, possessing an oscillator strength of 0.007. Additionally, the ST_7_ state appeared at 814 nm, with an oscillator strength of 0.065. It is worth noting that the SO-TDDFT calculations demonstrated a reasonable agreement with experimental spectra, as previously reported.^41^ In the NIR region, the calculated value of λ_max_ was 814 nm, exhibiting a red shift of merely 22 nm in comparison to the experimentally observed absorption peak at 792 nm.^41^ The ST_4_ state exhibited a red shift of 13 nm (0.020 eV) relative to the T_2_ state, while the ST_7_ state exhibited a blue shift of 8 nm (0.015 eV) relative to the S_1_ state. This shift was attributed to the fact that the energy difference (0.121 eV) between the excitation energies for S_1_ and T_2_ was smaller than the energy difference (0.175 eV) between the excitation energies for S_1_ and T_1_. Consequently, the ST_4_ state, mainly composed of the T_2_ state, borrowed oscillator strength from the S_1_ state due to the smaller denominator in eq 1. Furthermore, it is noteworthy that the absolute value of the matrix element ⟨Ψ_S_1__|*H*_SO_|Ψ_T_2__⟩ (0.0639 eV) was larger than that of ⟨Ψ_S_1__|*H*_SO_|Ψ_T_1__⟩ (0.0189 eV), as detailed in Table 3.

**Table 3:** Absolute Values of SOC Matrix Elements (eV) of DX-Ru-2Cl, DX-Ru-2Cl–N, and DX-Ru-2Cl–P(OMe)_3_ in DMF Solution

molecule	⟨Ψ_S_1__|*H*_SO_|Ψ_T_1__⟩	⟨Ψ_S_1__|*H*_SO_|Ψ_T_2__⟩	⟨Ψ_S_2__|*H*_SO_|Ψ_T_1__⟩	⟨Ψ_S_2__|*H*_SO_|Ψ_T_2__⟩
DX-Ru-2Cl	0.0189	0.0639	0.0642	0.0181
DX-Ru-2Cl–N	0.0668	0.0048	0.0062	0.0648
DX-Ru-2Cl–P(OMe)_3_	0.0146	0.0646	0.0650	0.0139

In the case of DX-Ru-2Cl–P(OMe)_3_, the lowest lying ST_4_ state resulted from SO interactions between S_1_ and T_2_, a scenario similar to that observed for the ST_4_ state of DX-Ru-2Cl. However, it is noteworthy that the ST_4_ state of DX-Ru-2Cl–P(OMe)_3_ exhibited an oscillator strength twice as large as that of the ST_4_ state in DX-Ru-2Cl. An examination of Table 3 reveals that the values of ⟨Ψ_S_1__|*H*_SO_|Ψ_T_2__⟩ for DX-Ru-2Cl–P(OMe)_3_ and DX-Ru-2Cl were comparable. Nevertheless, it is crucial to note that the energy difference (0.0629 eV) between S_1_ and T_2_ for DX-Ru-2Cl–P(OMe)_3_ was smaller in magnitude than that of DX-Ru-2Cl (0.121 eV). Consequently, the ST_4_ state of DX-Ru-2Cl–P(OMe)_3_ gained a higher intensity from the S_1_ state in comparison to the corresponding state in DX-Ru-2Cl.

DX-Ru-2Cl–N exhibited lower lying T_1_ (1024 nm) and T_2_ (946 nm) states compared to DX-Ru-2Cl (T_1_ = 930 nm and T_2_ = 894 nm) and DX-Ru-2Cl–P(OMe)_3_ (T_1_ = 914 nm and T_2_ = 848 nm). Consequently, the triplet states induced by the N-containing *pdn* ligand were positioned at lower energy levels than those associated with the P-containing ligand. The emergence of the ST_3_ state at 1036 nm, resulting from the SO interactions between the S_1_ and T_1_ states, is noteworthy. Despite its weak oscillator strength of only 0.002, it exhibited longer wavelength absorption in comparison to the other two complexes. This can be attributed to the fact that the ST_3_ state predominantly consists of the lowest lying T_1_ state, as opposed to the higher lying T_2_ states observed in DX-Ru-2Cl and DX-Ru-2Cl–P(OMe)_3_.

In summary, the phenyldimethoxyphosphine-derived ligands employed in the DX1-based Ru complexes did not substantially alter the SOC matrix elements as their contributions to electronic transitions were minimal. However, these ligands did influence the energy gap between the S_1_ state and the low-lying triplet states, resulting in SOC-activated transitions with varying oscillator strengths and absorption wavelengths.

### Electronic Structure and Absorption Spectra of DX1-Based Ru Complexes Coordinated with Halide or NCS^–^ Ligands

A prior theoretical investigation^23^ examined the capacity of halide ligands to induce SOC effects in DX1-based Ru complexes. In this study, we expanded our investigation to examine the influence of commonly employed NCS^–^ ligands on SOC effects, and we carried out a comparative analysis with the outcomes obtained using halide ligands. Table 4 provides an overview of the characteristics of the six frontier MOs. Figure S3 showcases the energy levels of these six frontier MOs and their corresponding isodensity plots for DX-Ru-2X (X = Br, I, or NCS) complexes. Significantly, the composition of the Ru 4d orbitals in the three HOMOs exhibited the following sequence: DX-Ru-2Cl > DX-Ru-2Br > DX-Ru-2I > DX-Ru-2NCS. In the case of DX-Ru-2Cl and DX-Ru-2Br complexes, the Ru 4d orbitals predominantly influenced all the three HOMOs. In contrast, the I^–^ ligands in the DX-Ru-2I complex and the NCS^–^ ligands in the DX-Ru-2NCS complex contributed more significantly to the three HOMOs than their respective Ru atoms. The π* orbitals of the anchoring ligand, *tctpy*, constitute the primary components of the three LUMOs. As a result, the low-energy singlet excitations observed in the DX-Ru-2Cl and DX-Ru-2Br complexes were likely attributed to metal-to-ligand charge transfer (^1^MLCT) from the Ru atom to the anchoring *tctpy* ligands. Conversely, the low-energy singlet excitations in the DX-Ru-2I and DX-Ru-2NCS complexes were predominantly driven by ligand-to-ligand charge transfer (^1^LLCT) from the I/NCS ligands to the anchoring *tctpy* ligand. Notably, among the DX-Ru-2X complexes, DX-Ru-2NCS exhibited the smallest HLG. This outcome stemmed from its higher energy HOMO and lower energy LUMO configurations.

**Table 4:** Characteristics of Frontier Orbitals of DX-Ru-2X (X = Br, I, or NCS)^a^^,^^b^

molecules	orbital	*E* (eV)	orbital character (%) of Ru	orbital character (%) of ligand (X)	orbital character (%) of *pdp*	orbital character (%) of *tctpy*
DX-Ru-2Br	L + 2	–2.605	0	0	0	93
	L + 1	–2.903	6d_*xy*_	0	0	88
	L	–3.357	8d_*yz*_	3p_*y*_	0	82
	H	–5.752	50d_*xy*_	30p_*x*_	0	9
	H – 1	–5.862	39d_*yz*_	39p_*z*_	0	7
	H – 2	–6.461	57d_*xz*_, 7d_*z*_^2^	9p_*x*_, 6p_*z*_	3	3
DX-Ru-2I	L + 2	–2.606	0	0	0	93
	L + 1	–2.910	6d_*xy*_	0	0	88
	L	–3.355	7d_*yz*_	5p_*y*_	0	82
	H	–5.711	41d_*xy*_	45p_*x*_	0	5
	H – 1	–5.778	29d_*yz*_	58p_*z*_	0	5
	H – 2	–6.332	33d_*xz*_, 5d_*z*_^2^	26p_*z*_, 22p_*x*_	0	2
DX-Ru-2NCS	L + 2	–2.651	0	0	0	93
	L + 1	–2.997	5d_*xy*_	0	0	89
	L	–3.451	7d_*yz*_	0	0	83
	H	–5.599	35d_*xy*_	51p_*x*_	0	2
	H – 1	–5.664	28d_*yz*_	54p_*z*_	0	6
	H – 2	–6.399	14d_*xz*_	60p_*x*_, 16p_*z*_	0	1

a *pdp* = [PPh(OMe)_2_] = phenyldimethoxyphosphine, *pdn* = [NPh(OMe)_2_] = phenyldimethoxynitryl *tmp* = [P(OMe)_3_] = trimethoxyphosphine, and *tctpy* = tricarboxyterpyridine.

b Orbital density below 5% is not listed.

**Figure 3:**
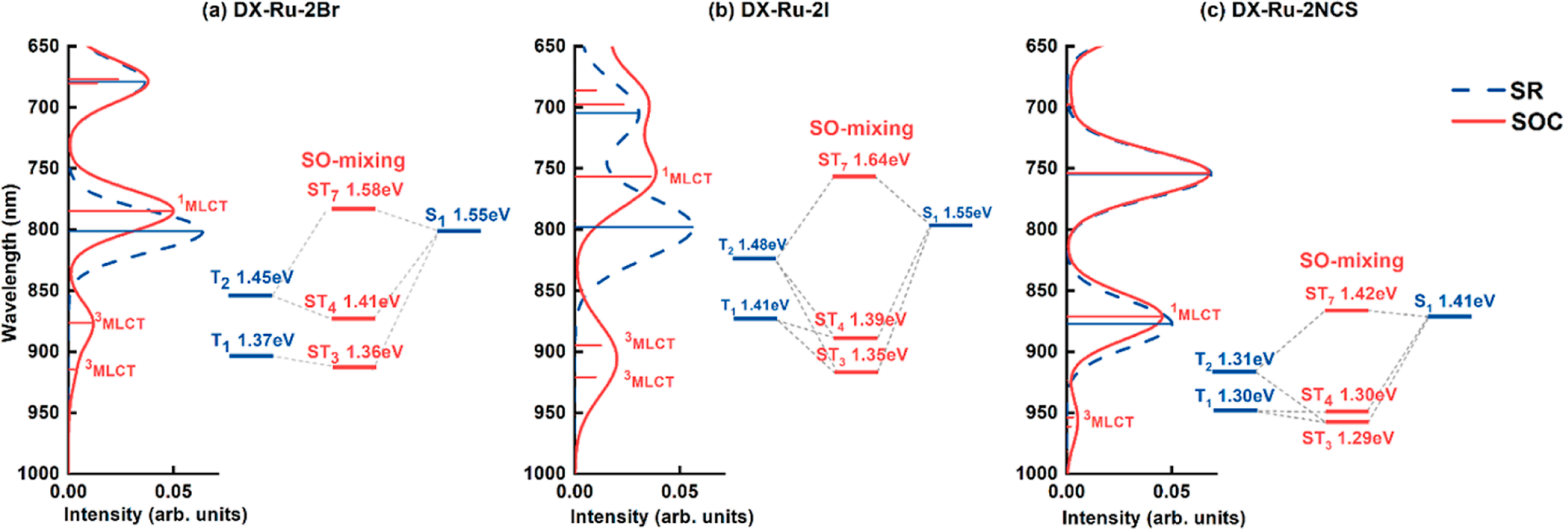
Simulated absorption spectra of (a) DX-Ru-2Br, (b) DX-Ru-2I, and (c) DX-Ru-2NCS complexes in DMF. Excitation energies and spectra calculated by SR-TDDFT are shown in blue, and excitation energies and spectra calculated by SO-TDDFT are shown in red. An energy diagram illustrating the SO mixing of singlet and triplet states, as calculated by SR-TDDFT, is presented alongside the spectra. The ST state represents the SO-mixed state of singlet and triplet excited states. The diagram specifically highlights the SO mixings that contribute to long-wavelength absorption.

Figure 3 presents the simulated absorption spectra in the NIR region for DX-Ru-2X complexes in DMF, obtained through both SR-TDDFT and SO-TDDFT calculations. The SOC transitions in the NIR region and their corresponding assignments, as computed by using SR-TDDFT and SO-TDDFT, are tabulated in Table 5. For a comprehensive overview, Figure S5 provides the complete SR-TDDFT and SO-TDDFT simulated absorption spectra for DX-Ru-2X complexes, and Tables S4–S6 furnish their respective assignments. The SR-TDDFT calculations revealed that the longest wavelength transitions, primarily stemming from HOMO–LUMO transitions, exhibited the following order: DX-Ru-2NCS (878 nm; *f* = 0.050) > DX-Ru-2Cl (822 nm; *f* = 0.073) > DX-Ru-2Br (802 nm; *f* = 0.064) > DX-Ru-2I (798 nm; *f* = 0.056). Notably, as we transitioned from DX-Ru-2Cl to DX-Ru-2Br and subsequently from DX-Ru-2Br to DX-Ru-2I, a decrease in the energy level of HOMO was observed. Consequently, employing halides with a larger atomic number as ligands led to a blue shift in the longest wavelength absorption.

**Table 5:** Calculated SOC-Induced Long-Wavelength Transitions of DX-Ru-2X (X = Br, I, and NCS) and Their Characters Contributed in Terms of SR Transitions^a^

perturbative SOC transition	transition assignments	SR contribution	SR composition
DX-Ru-2Br			
ST_3_: 1.36 eV (915 nm), *f* = 0.003	^3^MLCT	92% T_1_: 1.37 eV (902 nm), *f* = 0.000	87% (H→ L) 9% (H – 1 → L)
		4% S_1_: 1.55 eV (802 nm), *f* = 0.064	96% (H→ L)
ST_4_: 1.41 eV (877 nm), *f* = 0.012	^3^MLCT	78% T_2_: 1.45 eV (854 nm), *f* = 0.000	86% (H – 1 → L) 10% (H→ L)
		18% S_1_: 1.55 eV (802 nm), *f* = 0.064	96% (H→ L)
ST_7_: 1.58 eV (785 nm), *f* = 0.050	^1^MLCT	78% S_1_: 1.55 eV (802 nm), *f* = 0.064	96% (H→ L)
		20% T_2_: 1.45 eV (854 nm), *f* = 0.000	86% (H – 1 → L) 10% (H→ L)
DX-Ru-2I			
ST_3_: 1.35 eV (921 nm), *f* = 0.010	^3^LLCT	70% T_1_: 1.41 eV (881 nm), *f* = 0.000	86% (H→ L) 9% (H – 1 → L)
		15% S_1_: 1.55 eV (798 nm), *f* = 0.056	97% (H→ L)
		8% T_2_: 1.48 eV (837 nm), *f* = 0.000	85% (H – 1 → L) 10% (H→ L)
ST_4_: 1.39 eV (895 nm), *f* = 0.013	^3^LLCT	58% T_2_: 1.48 eV (837 nm), *f* = 0.000	85% (H – 1 → L) 10% (H→ L)
		19% S_1_: 1.55 eV (798 nm), *f* = 0.056	97% (H→ L)
		16% T_1_: 1.41 eV (881 nm), *f* = 0.000	86% (H→ L) 9% (H – 1 → L)
ST_7_: 1.64 eV (757 nm), *f* = 0.036	^1^LLCT	64% S_1_: 1.55 eV (798 nm), *f* = 0.056	97% (H→ L)
		30% T_2_: 1.48 eV (837 nm), *f* = 0.000	85% (H – 1 → L) 10% (H→ L)
DX-Ru-2NCS			
ST_3_: 1.29 eV (962 nm), *f* = 0.002	^3^LLCT	90% T_1_: 1.30 eV (956 nm), *f* = 0.000	85% (H→ L) 11% (H – 1 → L)
		6% T_2_: 1.31 eV (946 nm), *f* = 0.000	84% (H – 1 → L) 11% (H→ L)
		3% S_1_: 1.41 eV (878 nm), *f* = 0.050	98% (H→ L)
ST_4_: 1.30 eV (954 nm), *f* = 0.003	^3^LLCT	86% T_2_: 1.31 eV (946 nm), *f* = 0.000	84% (H – 1 → L) 11% (H→ L)
		8% T_1_: 1.30 eV (956 nm), *f* = 0.000	85% (H→ L) 11% (H – 1 → L)
		6% S_1_: 1.41 eV (878 nm), *f* = 0.050	98% (H→ L)
ST_7_: 1.42 eV (871 nm), *f* = 0.046	^1^LLCT	91% S_1_: 1.41 eV (878 nm), *f* = 0.050	98% (H→ L)
		8% T_2_: 1.31 eV (946 nm), *f* = 0.000	84% (H – 1 → L) 11% (H→ L)

a SR compositions by orbitals. Transitions with an oscillator strength lower than 0.001 are not shown here.

For the SO-TDDFT calculations, DX-Ru-2NCS featured the longest wavelength transition (ST_3_: 962 nm; *f* = 0.002) generated through SOC between the S_1_ and T_1_ states because the ST_3_ state for DX-Ru-2NCS was composed mainly of the lowest lying T_1_ state (among all of the DX-Ru-2X complexes); it had the longest wavelength absorption among these DX-Ru-2X complexes. The oscillator strength of the ST_3_ state for DX-Ru-2NCS was smaller than that (ST_4_; *f* = 0.007) for DX-Ru-2Cl. The Ru atom in DX-Ru-2NCS contributed less to the low-lying transitions than did the Ru atom of DX-Ru-2Cl; therefore, DX-Ru-2NCS had a smaller value of the SOC matrix element (Table 6). The value of ⟨Ψ_S_1__|*H*_SO_|Ψ_T_1__⟩ for DX-Ru-2NCS (0.0194 eV) was smaller than the value of ⟨Ψ_S_1__|*H*_SO_|Ψ_T_2__⟩ (0.0639 eV) for DX-Ru-2Cl. Furthermore, the energy difference between S_1_ and T_1_ for DX-Ru-2NCS (ca. 0.11 eV) was similar to that between S_1_ and T_2_ for DX-Ru-2Cl. These findings suggest that when ligands with smaller atomic numbers contribute less to low-lying transitions, allowing the larger atomic number Ru atom to dominate these transitions, it can result in the generation of strong SOC effects.

**Table 6:** Absolute Values of SOC Matrix Elements (eV) of DX-Ru-2X (X = Br, I, or NCS) in DMF Solution

molecule	⟨Ψ_S_1__|*H*_SO_|Ψ_T_1__⟩	⟨Ψ_S_1__|*H*_SO_|Ψ_T_2__⟩	⟨Ψ_S_2__|*H*_SO_|Ψ_T_1__⟩	⟨Ψ_S_2__|*H*_SO_|Ψ_T_2__⟩
DX-Ru-2Br	0.0420	0.0903	0.0911	0.0411
DX-Ru-2I	0.0714	0.1658	0.1676	0.0743
DX-Ru-2NCS	0.0194	0.0462	0.0449	0.0187

DX-Ru-2Br had three significant transitions that mixed singlet and spin-forbidden triplet excitations in the NIR region: 915 nm (ST_3_; *f* = 0.003), 877 nm (ST_4_; *f* = 0.012), and 785 nm (ST_7_; *f* = 0.050). The ST_3_ state which resulted from SO interactions between T_1_ and S_1_, consisted mainly of the T_1_ state (92%; *f* = 0.000); thus, it was a ^3^MLCT that borrowed intensity from the spin-allowed S_1_ state (4%; *f* = 0.064). In contrast, the intensity of the corresponding ST_3_ state for DX-Ru-2Cl was nearly negligible. The ST_3_ state of DX-Ru-2Br, on the other hand, arises from the SO mixing of the S_1_ state (comprising a 96% electron transition from H to L energy levels) and the T_1_ state (comprising 87% H to L and 9% H – 1 to L transitions). These states share similar electronic orbital components, resulting in comparable angular momentum. Consequently, the ST_3_ state exhibits an SOC matrix element of 0.0420 (eV) for ⟨Ψ_S_1__|*H*_SO_|Ψ_T_1__⟩, as the SO operator requires a difference in orbital angular momenta. This value is larger than that of DX-Ru-2Cl (0.0189 eV). This result highlights that the Br atom, with its larger orbit angular momentum compared to the Cl atom, can indeed enhance the SOC.

DX-Ru-2I had three transitions in the long-wavelength region: ST_3_ (921 nm; *f* = 0.010), ST_4_ (895 nm; *f* = 0.013), and ST_7_ (757 nm; *f* = 0.036). The ST_3_ state resulted from the SO interactions between T_1_ and S_1_. The T_1_ transition was mainly from the HOMO to the LUMO, while the ST_3_ state was mainly a ^3^LLCT. For the DX-Ru-2X (X = Cl, Br, or I) complexes, the longest wavelength transitions were dependent on the atomic number of the halides, following the order DX-Ru-2I (ST_3_: 921 nm; *f* = 0.010) > DX-Ru-2Br (ST_3_: 915 nm; *f* = 0.003) > DX-Ru-2Cl (ST_4_: 907 nm; *f* = 0.007). The relatively intense ST_3_ state for DX-Ru-2I resulted from the largest absolute values of the SOC matrix element ⟨Ψ_S_1__|*H*_SO_|Ψ_T_2__⟩, which reached up to 0.16 eV (Table 6). Moreover, the longest wavelength of the ST_3_ state for DX-Ru-2I was due to the fact that it was composed mainly of the low-lying T_1_ state. These findings indicate that both I and Ru atoms, characterized by larger atomic numbers and consequently possessing significant orbital angular momentum, exhibit the capacity to induce SO interactions.

### Electronic Structures and Absorption Spectra of BD-Ru-3X Complexes with Three Identical Halides or NCS^–^ Ligands

In the preceding section, we observed that I^–^ employed as ligands in DX1-based complexes actively participated in low-lying transitions, thereby augmenting SOC effects to activate spin-forbidden transitions. In this section, we investigate the impact of three identical halides or NCS^–^ ligands on the absorption spectra of BD-Ru-3X (X = Cl, Br, I, or NCS) complexes. Table 7 provides an overview of the characteristics of the six frontier MOs for the BD-Ru-3X complexes, while Figure S5 presents their respective isodensity plots. It is noteworthy that the π* orbitals of the anchoring ligand, *tctpy*, predominantly govern the configuration of the three LUMOs. Comparing these complexes to their Ru-based counterparts, DX-Ru-2X, reveals notable distinctions in the contributions of ancillary ligands to various MOs. Specifically, the orbitals of the ancillary ligands in each BD-Ru-3X complex made more substantial contributions to H – 2 compared to their counterparts in DX-Ru-2X complexes. Conversely, the ancillary ligand orbitals in BD-Ru-3X complexes exhibited reduced contributions to HOMO and H – 1 relative to DX-Ru-2X complexes. For BD-Ru-3I, it is noteworthy that the Ru 4d orbitals contributed more significantly to the three HOMOs than those observed in DX-Ru-2I. Consequently, the low-lying transitions in BD-Ru-3I were more likely attributable to MLCT. On the other hand, in the case of BD-Ru-3NCS, the NCS ligands made more substantial contributions to the three HOMOs compared to the Ru atom itself. Consequently, the long-wavelength transitions in BD-Ru-3NCS were primarily associated with LLCT.

**Table 7:** Characteristics of Frontier Orbitals of BD-Ru-3X (X = Cl, Br, I, or NCS)^a^^,^^b^

molecules	orbital	*E* (eV)	orbital character (%) of Ru	orbital character (%) of ligand (X)	orbital character (%) of *tctpy*
BD-Ru-3Cl	L + 2	–2.520	0	0	92
	L + 1	–2.704	8d_*xy*_	0	87
	L	–3.121	14d_*yz*_	0	77
	H	–5.549	49d_*yz*_	16p_*z*_, 5p_*y*_	19
	H – 1	–5.569	58d_*xy*_	22p_*x*_	15
	H – 2	–5.722	63d_*xz*_	20p_*x*_	0
BD-Ru-3Br	L + 2	–2.542	0	0	92
	L + 1	–2.731	8d_*xy*_	0	87
	L	–3.157	13d_*yz*_	0	77
	H	–5.598	51d_*xy*_	27p_*x*_	14
	H – 1	–5.605	44d_*yz*_	19p_*z*_, 6p_*y*_	15
	H – 2	–5.717	57d_*xz*_	27p_*x*_	0
BD-Ru-3I	L + 2	–2.540	0	0	92
	L + 1	–2.740	8d_*xy*_	0	87
	L	–3.170	13d_*yz*_	0	77
	H	–5.570	44d_*xy*_	40p_*x*_	9
	H – 1	–5.614	41d_*yz*_	30p_*z*_, 7p_*y*_	11
	H – 2	–5.651	45d_*xz*_	42p_*x*_	0
BD-Ru-3NCS	L + 2	–2.620	0	0	92
(original BD)	L + 1	–2.880	6d_*xz*_	0	88
	L	–3.317	11d_*yz*_	0	79
	H	–5.476	31d_*yz*_	37p_*y*_, 10p_*z*_	10
	H – 1	–5.515	35d_*xz*_	52p_*x*_	6
	H – 2	–5.720	38d_*xy*_	50p_*x*_	0

a *pdp* = [PPh(OMe)_2_] = phenyldimethoxyphosphine, *pdn* = [NPh(OMe)_2_] = phenyldimethoxynitryl, *tmp* = [P(OMe)_3_] = trimethoxyphosphine, and *tctpy* = tricarboxyterpyridine.

b Orbital density below 5% is not listed.

**Figure 4:**
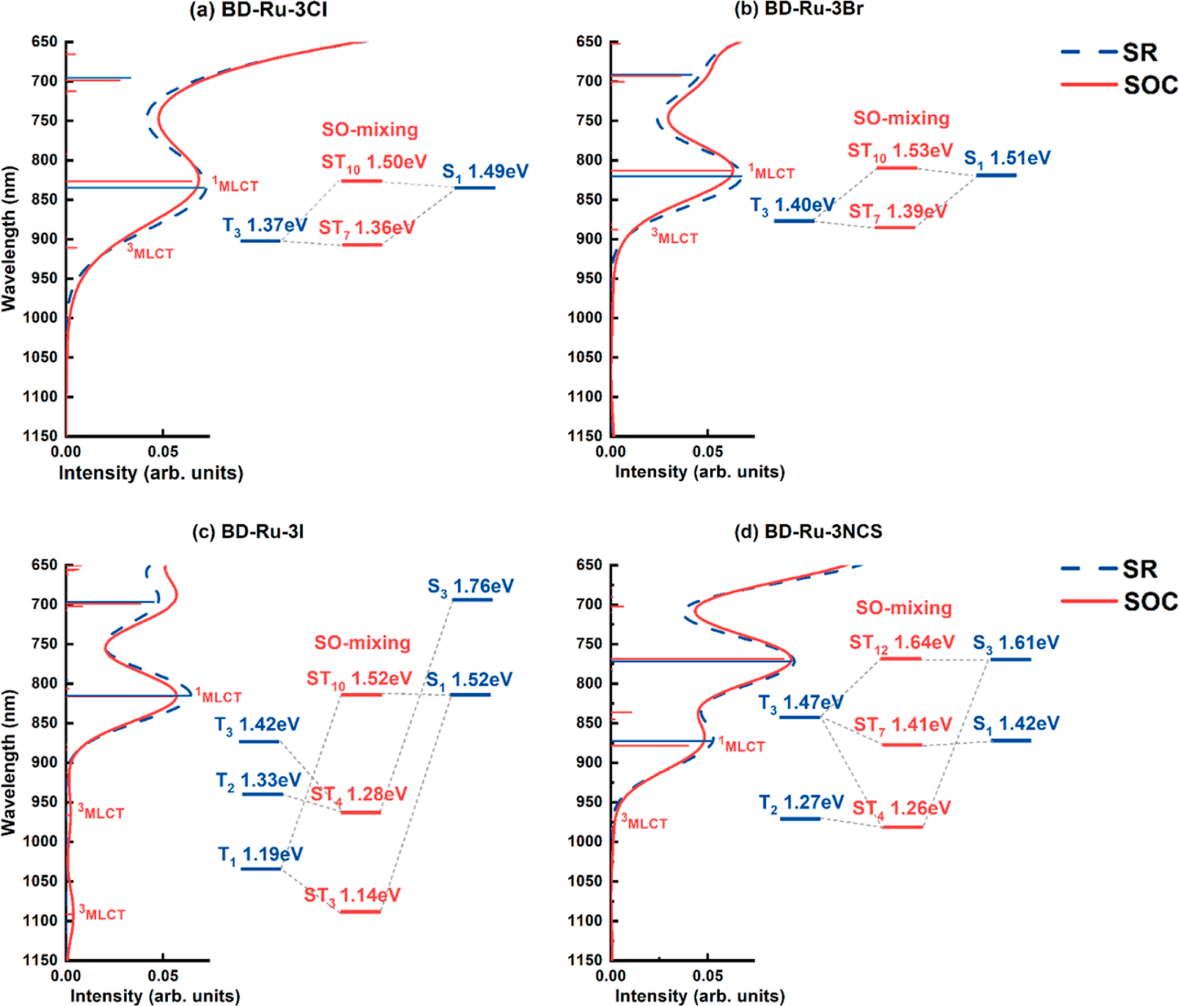
Simulated absorption spectra of (a) BD-Ru-3Cl, (b) BD-Ru-3Br, (c) BD-Ru-3I, and (d) BD-Ru-3NCS complexes in DMF. Excitation energies and spectra calculated by SR-TDDFT are shown in blue, and excitation energies and spectra calculated by SO-TDDFT are shown in red. An energy diagram illustrating the SO mixing of singlet and triplet states, as calculated by SR-TDDFT, is presented alongside the spectra. The ST state represents the SO-mixed state of singlet and triplet excited states. The diagram specifically highlights the SO mixings that contribute to long-wavelength absorption.

Figure 4 presents the simulated absorption spectra of the BD-Ru-3X complexes within the NIR region, calculated by both SR-TDDFT and SO-TDDFT. Table 8 provides a comprehensive summary of the significant SO-induced transitions and their associated characteristics in terms of SR transitions. For a complete overview, Figure S3 showcases the full SR- and SO-TDDFT-simulated absorption spectra (λ > 400 nm) of the BD-Ru-3X complexes, while Tables S7–S9 compile their respective assignments. Notably, the SO-TDDFT-simulated spectra of BD-Ru-3NCS (Figure S3) demonstrated qualitative consistency with the experimental spectra of BD, with the calculated λ_max_ exhibiting a 70 nm blue shift compared to the experimentally observed λ_max_ peak at 599 nm.^18,20^ From the SR-TDDFT calculations, it was determined that among the BD-Ru-3X complexes, BD-Ru-3NCS exhibited the longest wavelength absorption (S1: 872 nm; *f* = 0.051). The order of longest wavelength absorptions for the BD-Ru-3X complexes is as follows: BD-Ru-3NCS > BD-Ru-3Cl (834 nm; *f* = 0.071) > BD-Ru-3Br (820 nm; *f* = 0.067) > BD-Ru-3I (815 nm; *f* = 0.056). This trend mirrored the values observed for the DX1-based Ru complexes. Notably, except for BD-Ru-3NCS, the Ru complexes featuring three identical halides as ligands exhibited lower lying S_1_ transitions compared to their corresponding DX1-based Ru complexes featuring two halides as ligands.

**Table 8:** Calculated SOC-Induced Long-Wavelength Transitions of BD-Ru-3X (X = Cl, Br, I, or NCS) and Their Characters Contributed in Terms of SR Transitions^a^

perturbative SOC transition	transition assignments	SR contribution	SR composition
BD-Ru-3Cl			
ST_7_: 1.36 eV (910 nm), *f* = 0.005	^3^MLCT	89% T_3_: 1.37 eV (907 nm), *f* = 0.000	95% (H – 2→ L)
		8% S_1_: 1.49 eV (834 nm), *f* = 0.071	92% (H – 1 → L)
ST_10_: 1.50 eV (826 nm), *f* = 0.065	^1^MLCT	91% S_1_: 1.49 eV (834 nm), *f* = 0.071	92% (H – 1 → L)
		8% T_3_: 1.37 eV (907 nm), *f* = 0.000	95% (H – 2→L)
BD-Ru-3Br			
ST_7_: 1.39 eV (887 nm), *f* = 0.003	^3^MLCT	90% T_3_: 1.40 eV (888 nm), *f* = 0.000	95% (H – 2→ L)
		5% S_1_: 1.51 eV (820 nm), *f* = 0.067	94% (H → L)
ST_10_: 1.53 eV (812 nm), *f* = 0.062	^1^MLCT	92% S_1_: 1.51 eV (820 nm), *f* = 0.067	94% (H → L)
		6% T_3_: 1.40 eV (888 nm), *f* = 0.000	95% (H – 2 → L)
BD-Ru-3I			
ST_3_: 1.14 eV (1091 nm), *f* = 0.004	^3^MLCT	86% T_1_: 1.19 eV (1046 nm), *f* = 0.000	89% (H – 1 → L) 5% (H – 1 → L + 2)
		6% S_1_: 1.52 eV (815 nm), *f* = 0.056	95% (H → L)
ST_4_: 1.28 eV (966 nm), *f* = 0.002	^3^MLCT	82% T_2_: 1.33 eV (933 nm), *f* = 0.000	94% (H → L)
		12% T_3_: 1.42 eV (874 nm), *f* = 0.000	92% (H – 2 → L)
		5% S_3_: 1.78 eV (696 nm), *f* = 0.045	85% (H – 1 → L) 12% (H → L + 1)
ST_10_: 1.52 eV (815 nm), *f* = 0.056	^1^MLCT	87% S_1_: 1.52 eV (815 nm), *f* = 0.056	95% (H → L)
		5% T_1_: 1.19 eV (1046 nm), *f* = 0.000	89% (H – 1 → L)5% (H – 1 → L + 2)
BD-Ru-3NCS			
ST_4_: 1.26 eV (983 nm), *f* = 0.001	^3^LLCT	95% T_2_: 1.27 eV (975 nm), *f* = 0.000	97% (H – 1 → L)
		3% T_3_: 1.47 eV (846 nm), *f* = 0.000	94% (H – 2→ L)
			5% (H – 7 → L)
		2% S_3_: 1.61 eV (771 nm), *f* = 0.093	89% (H → L) 9% (H – 1 → L + 1)
ST_7_: 1.41 eV (878 nm), *f* = 0.040	^1^LLCT	80% S_1_: 1.42 eV (872 nm), *f* = 0.051	97% (H – 1 → L)
		19% T_3_: 1.47 eV (846 nm), *f* = 0.000	94% (H – 2→ L) 5% (H-7→ L)
ST_12_: 1.61 eV (768 nm), *f* = 0.089	^1^LLCT	96% S_3_: 1.61 eV (771 nm), *f* = 0.093	89% (H→ L) 9% (H – 1→ L + 1)
		2% T_3_: 1.47 eV (846 nm), *f* = 0.000	94% (H – 2→ L) 5% (H – 7→ L)

a SR compositions by orbitals. Transitions with an oscillator strength lower than 0.001 are not shown here.

The SR-TDDFT calculations revealed that BD-Ru-3Cl featured one intense peak in the NIR region, S_1_ (834 nm; *f* = 0.071), and one intense peak in the visible region, S_3_ (695 nm; *f* = 0.033). The major composition of S_1_ was the H – 1→ L (92%) transition; for S_3_, it was the H→ L (77%) transition. Through SO interactions, T_3_ and S_1_ generated ST_7_ (910 nm; *f* = 0.005) and ST_10_ (826 nm; *f* = 0.065) states. The ST_7_ state was composed mainly of the T_3_ state and, thus, it was a ^3^MLCT. The SR-TDDFT calculations revealed that BD-Ru-3Br had one intense peak in the NIR region, at 820 nm (S_1_ state; *f* = 0.067), and one intense peak in the visible region, at 691 nm (S_3_ state; *f* = 0.042). The transition of the S_1_ state consisted mainly of a H → L (94%) transition. The transition of the S_3_ state arose from H – 1 → L (81%) and H → L + 1 (17%) transitions. The SO-TDDFT calculations revealed two significant transitions mixing singlet and triplet excitations in the NIR region: at 887 (ST_7_; *f* = 0.003) and 812 nm (ST_10_; *f* = 0.062). The lowest excited state ST_7_ resulted from SO interactions between T_3_ and S_1_ and was composed mainly of the T_3_ state (88%; *f* = 0.000); thus, it was a ^3^MLCT that borrowed intensity from the spin-allowed S_1_ state (5%; *f* = 0.067). The ST_10_ state arose from SO interactions between S_1_ and T_3_; it was composed mainly of the S_1_ state (92%; *f* = 0.067). Next, we examined the simulated spectra of the BD-Ru-3I complexes. The SR-TDDFT calculations revealed that BD-Ru-3I had one intense peak at 815 nm (S_1_ state; *f* = 0.056) that arose mainly from the H → L (95%) transition. SO-TDDFT calculations revealed that ST_3_ (1091 nm; *f* = 0.004) and ST_10_ (815 nm. *f* = 0.056) states were generated through SO interactions between the S_1_ and T_1_ states. Collectively, the excitation energy of the ST_3_ state in BD-Ru-3I was lower than that in the other BD-Ru-3X complexes. This is attributed to the fact that it primarily consisted of the lower lying T_1_ state. However, it is noteworthy that the oscillator strength of the ST_3_ state in BD-Ru-3I is similar to that of the ST_7_ states in BD-Ru-3Cl and BD-Ru-3Br, despite the significantly higher absolute value of ⟨Ψ_S_1__|*H*_SO_|Ψ_T_1__⟩ that generates the ST_3_ state in BD-Ru-3I (reaching up to 0.1271 eV, approximately double the values of ⟨Ψ_S_1__|*H*_SO_|Ψ_T_3__⟩ for generating the ST_7_ states in BD-Ru-3Cl and BD-Ru-3Br, as indicated in Table 9). Our analysis revealed that |*E*_S_1__ – *E*_T_1__|| for BD-Ru-3I (up to 0.33 eV) was 3 times larger than the values of |*E*_S_1__ – *E*_T_1__| (approximately 0.1 eV) for BD-Ru-3Cl and BD-Ru-3Br. This larger value of |*E*_S_1__ – *E*_T_1__|for BD-Ru-3I resulted in a reduction of its SOC effects as indicated in eq 1.

**Table 9:** Absolute Values of SOC Matrix Elements (eV) of BD-Ru-3X (X = Cl, Br, I, or NCS) in DMF Solution

molecule	⟨Ψ_S_1__|*H*_SO_|Ψ_T_1__⟩	⟨Ψ_S_1__|*H*_SO_|Ψ_T_3__⟩	⟨Ψ_S_3__|*H*_SO_|Ψ_T_2__⟩	⟨Ψ_S_3__|*H*_SO_|Ψ_T_3__⟩
BD-Ru-3Cl	0.0670	0.0624	0.0687	0.0079
BD-Ru-3Br	0.0866	0.0588	0.0895	0.0119
BD-Ru-3I	0.1271	0.0533	0.1445	0.0106
BD-Ru-3NCS	0.0030	0.0470	0.0029	0.0395

SR-TDDFT calculations revealed that BD-Ru-3NCS had two major intense peaks in the NIR region, at 872 nm (S_1_ state; *f* = 0.051) and 771 nm (S_3_ state; *f* = 0.093), and one major intense peak in the visible region, at 641 nm (S_4_ state; *f* = 0.125). The major composition of S_1_ arose from the H – 1→ L transition (97%); S_3_ arose mainly from the H → L transition (89%), while S_4_ arose mainly from the H → L + 1 transition (98%). The weak ST_4_ state (983 nm; *f* = 0.001), located in the NIR region, resulted from SO interactions among T_2_, T_3_, and S_3_, and was composed mainly of the T_2_ state (95%; *f* = 0.000); thus, it was a ^3^LLCT that borrowed intensity from the spin-allowed S_3_ state (2%; *f* = 0.093). The ST_4_ state for BD-Ru-3NCS was very weak, mainly because of its low value of ⟨Ψ_S_3__|*H*_SO_|Ψ_T_2__⟩ of 0.0029 eV (Table 9). As revealed in the previous MO analysis, the NCS ligands contributed more to the three occupied MOs and, thus, the Ru 4d orbitals contributed less, leading to a smaller SOC matrix.

### Effects of Os Atom on the Absorption Spectra of DX1-Based Os Complexes

Certain Ru complexes, such as DX1, have demonstrated effective SOC effects that result in longer wavelength absorptions that are suitable for applications in DSSCs. Although Os atoms possess larger orbital angular momentum than Ru atoms, making them suitable for inducing longer wavelength absorptions in the NIR through SOC, Os complexes have received limited attention as sensitizers in DSSCs.^15−21,42^ Considering this, we investigated the impact of Os atoms with larger atomic numbers on the activation of triplet states through SOC. The characteristics of the six frontier MOs for DX1-based Os complexes, DX-Os-2X (X = Cl, Br, I, or NCS), are summarized in Table 10, while their isodensity plots are presented in Figure S7. Notably, the three LUMOs in the DX-Os-2X complexes were primarily governed by the π* orbitals of the *tctpy* moiety. In the case of DX-Os-2Cl, the three HOMOs were predominantly influenced by the 5d orbitals. However, this influence diminished as one moved from DX-Os-2Cl to DX-Os-2Br and further to DX-Os-2I. Conversely, for DX-Os-2NCS, the NCS ligands made more substantial contributions to the three HOMOs compared with the Os 5d orbitals. Notably, DX-Os-2NCS exhibited the smallest HLG among the DX-Os-2X complexes. It is worth mentioning that each DX-Os-2X complex possessed an HLG smaller than that of its DX1-based Ru counterpart.

**Table 10:** Characteristics of Frontier Orbitals of DX-Os-2X (X = Cl, Br, I, or NCS)^a^^,^^b^

molecules	orbital	*E* (eV)	orbital character (%) of Os	orbital character (%) of ligand (X)	orbital character (%) of *pdp*	orbital character (%) of *tctpy*
DX-Os-2Cl	L + 2	–2.615	0	0	0	93
	L + 1	–2.819	10d_*xy*_	0	0	85
	L	–3.352	11d_*yz*_	0	0	78
	H	–5.537	54d_*xy*_	22p_*x*_	0	19
	H – 1	–5.753	46d_*yz*_	28p_*z*_	0	13
	H – 2	–6.397	67d_*xz*_, 6d_*z*_^2^	0	5	0
DX-Os-2Br	L + 2	–2.630	0	0	0	93
	L + 1	–2.836	9d_*xy*_	0	0	85
	L	–3.366	10d_*yz*_	0	0	79
	H	–5.567	50d_*xy*_	27p_*x*_	0	17
	H – 1	–5.758	39d_*yz*_	36p_*z*_	0	11
	H – 2	–6.348	61d_*xz*_, 6d_*z*_^2^	6p_*x*_	5	0
DX-Os-2I	L + 2	–2.628	0	0	0	93
	L + 1	–2.843	9d_*xy*_	0	0	86
	L	–3.367	9d_*yz*_	0	0	79
	H	–5.550	44d_*xy*_	38p_*x*_	0	15
	H – 1	–5.696	32d_*yz*_	53p_*z*_	0	7
	H – 2	–6.278	52d_*xz*_, 5d_*z*_^2^	18p_*x*_, 6p_*z*_	0	0
DX-Os-2NCS	L + 2	–2.679	0	0	0	93
	L + 1	–2.955	7d_*xy*_	0	0	86
	L	–3.465	10d_*yz*_	0	0	78
	H	–5.415	36d_*xy*_	47p_*x*_	0	10
	H – 1	–5.547	28d_*yz*_	48p_*z*_	0	9
	H – 2	–6.402	29d_*xz*_	48p_*x*_, 7p_*z*_	0	0

a *pdp* = [PPh(OMe)_2_] = phenyldimethoxyphosphine, *pdn* = [NPh(OMe)_2_] = phenyldimethoxynitryl, *tmp* = [P(OMe)_3_] = trimethoxyphosphine, and *tctpy* = tricarboxyterpyridine.

b Orbital density below 5% is not listed.

**Figure 5:**
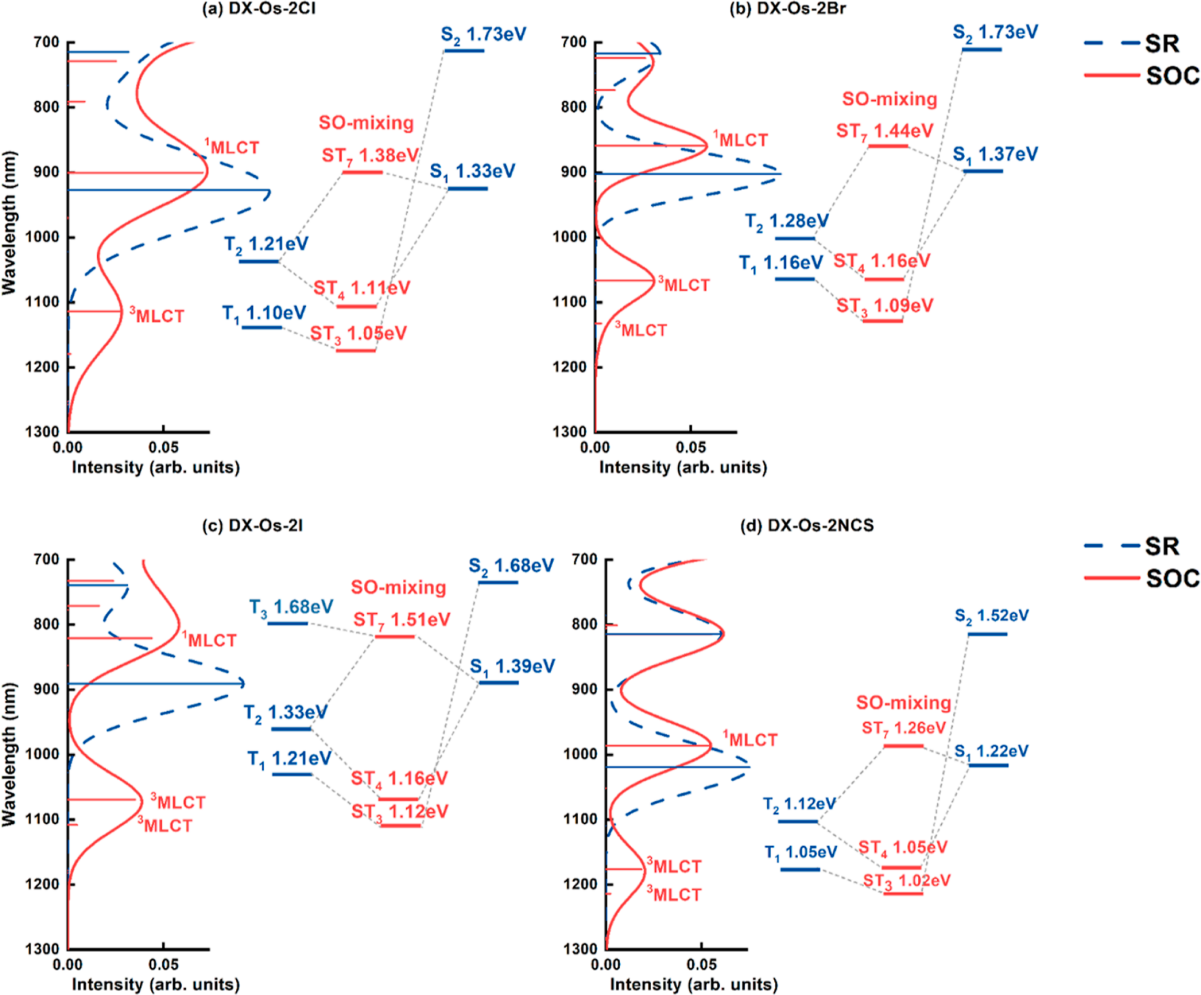
Simulated absorption spectra of (a) DX-Os-2Cl, (b) DX-Os-2Br, (c) DX-Os-2I, and (d) DX-Os-2NCS complexes in DMF. Excitation energies and spectra calculated by SR-TDDFT are shown in blue, and excitation energies and spectra calculated by SO-TDDFT are shown in red. An energy diagram illustrating the SO mixing of singlet and triplet states, as calculated by SR-TDDFT, is presented alongside the spectra. The ST state represents the SO-mixed state of singlet and triplet excited states. The diagram specifically highlights the SO mixings that contribute to long-wavelength absorption.

Figure 5 presents the simulated absorption spectra of the DX-Os-2X (X = Cl, Br, I, or NCS) complexes computed using both SR-TDDFT and SO-TDDFT. Table 11 provides an overview of the long-wavelength transitions induced by SO interactions. For a comprehensive analysis, Figure S8 showcases the complete simulated absorption spectra (λ > 400 nm), calculated using SR- and SO-TDDFT, for the DX-Os-2X complexes, while Tables S11–S14 compile their respective assignments. Through SR-TDDFT calculations, it was determined that the longest wavelength transitions (S_1_) among the DX-Os-2X complexes followed the order DX-Os-2NCS (1020 nm; *f* = 0.075) > DX-Os-2Cl (929 nm; *f* = 0.105) > DX-Os-2Br (903 nm; *f* = 0.097) > DX-Os-2I (891 nm; *f* = 0.091). This trend aligned with that observed for the DX1-based Ru counterparts. However, it is noteworthy that the energy of the S_1_ state for each DX-Os-2X complex was significantly lower than that of its Ru counterpart. Consequently, the S_1_ transition energies for the DX-Os-2X complexes fell within the NIR region.

**Table 11:** Calculated SOC-Induced Long-Wavelength Transitions of DX-Os-2X (X = Cl, Br, I, or NCS) and Their Characters Contributed in Terms of SR Transitions^a^

perturbative SOC transition	transition assignments	SR contribution	SR composition
DX-Os-2Cl			
ST_3_: 1.05 eV (1180 nm), *f* = 0.002	^3^MLCT	92% T_1_: 1.10 eV (1125 nm), *f* = 0.000	97% (H → L)
		4% S_2_: 1.73 eV (716 nm), *f* = 0.032	77% (H – 1 → L) 22% (H→ L + 1)
ST_4_: 1.11 eV (1114 nm), *f* = 0.027	^3^MLCT	70% T_2_: 1.21 eV (1024 nm), *f* = 0.000	92% (H – 1 → L)
		26% S_1_: 1.33 eV (929 nm), *f* = 0.105	97% (H → L)
ST_7_: 1.38 eV (900 nm), *f* = 0.070	^1^MLCT	67% S_1_: 1.33 eV (929 nm), *f* = 0.105	97% (H → L)
		28% T_2_: 1.21 eV (1024 nm), *f* = 0.000	92% (H – 1 → L)
DX-Os-2Br			
ST_3_: 1.09 eV (1133 nm), *f* = 0.003	^3^MLCT	88% T_1_: 1.16 eV (1073 nm), *f* = 0.000	95% (H → L)
		5% S_2_: 1.73 eV (717 nm), *f* = 0.034	83% (H – 1 → L) 16% (H → L + 1)
ST_4_: 1.16 eV (1066 nm), *f* = 0.030	^3^MLCT	62% T_2_: 1.28 eV (966 nm), *f* = 0.000	91% (H – 1 → L) 10% (H → L)
		31% S_1_: 1.37 eV (903 nm), *f* = 0.097	97% (H → L)
ST_7_:1.44 eV (860 nm), *f* = 0.058	^1^MLCT	60% S_1_: 1.37 eV (903 nm), *f* = 0.097	97% (H → L)
		34% T_2_: 1.28 eV (966 nm), *f* = 0.000	91% (H – 1 → L) 10% (H → L)
DX-Os-2I			
ST_3_: 1.12 eV (1109 nm), *f* = 0.005	^3^MLCT	84% T_1_: 1.21 eV (1028 nm), *f* = 0.000	96% (H → L)
		10% S_2_: 1.68 eV (739 nm), *f* = 0.031	90% (H – 1 → L) 9% (H→ L + 1)
ST_4_: 1.16 eV (1069 nm), *f* = 0.035	^3^LLCT	54% T_2_: 1.33 eV (931 nm), *f* = 0.000	92% (H – 1 → L)
		38% S_1_: 1.39 eV (891 nm), *f* = 0.091	98% (H → L)
ST_7_: 1.51 eV (821 nm), *f* = 0.044	^1^MLCT	47% S_1_: 1.39 eV (891 nm), *f* = 0.091	98% (H → L)
		38% T_2_: 1.33 eV (931 nm), *f* = 0.000	92% (H – 1 → L)
		12% T_3_: 1.68 eV (736 nm), *f* = 0.000	90% (H → L + 1)
DX-Os-2NCS			
ST_3_: 1.02 eV (1214 nm), *f* = 0.003	^3^LLCT	94% T_1_: 1.05 eV (1180 nm), *f* = 0.000	97% (H → L)
		3% S_2_: 1.52 eV (815 nm), *f* = 0.060	86% (H – 1 → L) 3% (H→ L + 1)
ST_4_: 1.05 eV (1177 nm), *f* = 0.019	^3^LLCT	72% T_2_: 1.12 eV (1109 nm), *f* = 0.000	94% (H – 1 → L)
		25% S_1_: 1.22 eV (1020 nm), *f* = 0.075	98% (H → L)
ST_7_: 1.26 eV (986 nm), *f* = 0.055	^1^LLCT	73% S_1_: 1.22 eV (1020 nm), *f* = 0.075	98% (H → L)
		26% T_2_: 1.12 eV (1109 nm), *f* = 0.000	94% (H – 1 → L)

a SR compositions by orbitals. Transitions with an oscillator strength lower than 0.001 are not shown here.

For the DX-Os-2X complexes, we found that the Os atoms induced SOC effects stronger than those of the Ru atoms. Two triplet states were activated with absorption wavelengths greater than 1000 nm: one (ST_3_), located in the longer wavelength region, was relatively weaker in intensity than the other (ST_4_), located at a shorter wavelength. The ST_3_ state, which was composed mainly of the T_1_ state, resulted from SOC between the T_1_ and S_2_ states; the ST_4_ state, which was composed mainly of the T_2_ state, resulted from SOC between the T_2_ and S_1_ states. The ST_3_ state had a longer wavelength absorption than that of the ST_4_ state because the energy of the main component (T_1_) of the ST_3_ state was lower than that of the main component (T_2_) of the ST_4_ state. The stronger absorption of the ST_4_ state, relative to that of the ST_3_ state, can be understood by considering the smaller energy difference between the S_1_ and T_2_ states (the SOC yielding the ST_4_ state) than that between the T_1_ and S_1_ states (the SOC yielding the ST_3_ state).

Table 12 lists the absolute values of the SOC matrix elements of the DX-Os-2X complexes. For the ⟨Ψ_S_1__|*H*_SO_|Ψ_T_2__⟩ SOC matrix elements, the absolute values for the DX-Os-2X complexes were significantly larger than those for the corresponding DX-Ru-2X complexes. Moreover, the absolute value of the ⟨Ψ_S_1__|*H*_SO_|Ψ_T_2__⟩ SOC matrix element increased upon proceeding from DX-Os-2Cl to DX-Os-2Br and from DX-Os-2Br to DX-Os-2I. In particular, for the DX-Os-2I complex, the absolute value of the ⟨Ψ_S_1__|*H*_SO_|Ψ_T_2__⟩ SOC matrix element reached up to 0.23 eV. Hence, ligands characterized by a substantial atomic number, such as I^–^, in combination with a high atomic number Os atom, facilitate SO interactions. Furthermore, due to the significant SOC matrix elements in the DX-Os-2X complexes, SOC effectively activates two states with a considerable energy difference, exemplified by ST_3_. For DX-Os-2Cl, the energy gap between the T_1_ and S_2_ states extended to 0.63 eV, surpassing the value of 0.12 eV between the T_2_ and S_1_ states.

**Table 12:** Absolute Values of SOC Matrix Elements (eV) of DX-Os-2X (X = Cl, Br, I, or NCS) in DMF Solution

molecule	⟨Ψ_S_1__|*H*_SO_|Ψ_T_2__⟩	⟨Ψ_S_1__|*H*_SO_|Ψ_T_3__⟩	⟨Ψ_S_2__|*H*_SO_|Ψ_T_1__⟩
DX-Os-2Cl	0.1488	0.0527	0.1679
DX-Os-2Br	0.1710	0.0522	0.1862
DX-Os-2I	0.2349	0.0566	0.2473
DX-Os-2NCS	0.1136	0.0339	0.1209

## Conclusions

We employed TDDFT coupled with SO interactions to conduct simulations of the absorption spectra, with a particular focus on long-wavelength absorption and triplet transitions activated through SOC, in Ru/Os dyes based on the DX1 framework and BD-like Ru dyes featuring halide and NCS^–^ as ancillary ligands. Our SR-TDDFT calculations unveiled that the nature of the ancillary ligands had a substantial impact on the longest wavelength spin-allowed absorption: Ru/Os complexes containing NCS^–^ ligands exhibited longer wavelength S_1_ transitions compared to their counterparts with halides as ligands. In the case of DX-Ru-2X, DX-Os-2X, and BD-Ru-3X complexes, the presence of high atomic number halide ligands led to a blue shift in the S_1_ transition. Within a given series of Ru or Os complexes, the wavelength of the S_1_ transition followed the order NCS > Cl > Br > I. Moreover, when comparing Ru and Os counterparts with identical ligands, the Os complex consistently exhibited a longer wavelength absorption of the S_1_ transition than its Ru counterpart. Notably, the DX-Os-2NCS complex exhibited an S_1_ transition wavelength exceeding 1000 nm (1020 nm; *f* = 0.075), whereas its counterpart in the Ru series, DX-Ru-2NCS, exhibited an S_1_ transition at 878 nm.

The absorption spectra analysis of the investigated Ru/Os dyes elucidated the pivotal role played by ancillary ligands in activating spin-forbidden triplet transitions through SO interactions. Specifically, it was observed that the contributions of ancillary ligands to the high-energy occupied MOs are of paramount importance in facilitating these transitions. Notably, ancillary ligands characterized by a higher atomic number, corresponding to larger orbital angular momentum, exhibited a substantial influence on the high-energy occupied MOs. In contrast, ancillary ligands with lower atomic numbers made relatively modest contributions, thereby amplifying the influence of the high atomic number of Ru/Os atoms on these transitions, leading to pronounced SOC effects. The specific contributions of various ligands were found to vary. Cl^–^ ligands in the DX-Ru-2Cl complex made relatively minor contributions, while phenyldimethoxyphosphine ligands exhibited negligible contributions to the high-energy occupied MOs, resulting in the dominance of the high atomic number Ru atom in driving the transitions and reinforcing SO interactions. Conversely, the NCS^–^ ligands in the DX-Ru-2NCS complexes contributed significantly to the high-energy occupied MOs, thereby diminishing the role of the high atomic number central metals and consequently weakening the SO interactions. Furthermore, it was observed that the SOC effect in BD-Ru-3NCS was notably weak due to the small values of the SOC matrix elements. In contrast, the orbitals of high atomic number I^–^ ligands, such as those in DX-Ru-2I, in conjunction with the high atomic number Ru atoms, exhibited significant contributions to the high-energy occupied MOs, thus enhancing the overall SO interactions.

The results obtained from SO-TDDFT calculations have provided valuable insights into the nature of electronically excited states activated by SOC interactions in the studied systems. It was observed that the SOC-activated transitions were predominantly associated with triplet states. Consequently, the wavelength corresponding to the longest transition activated by SOC interactions was primarily determined by the energy of the lower lying triplet states, as determined by SR-TDDFT calculations. In this context, it is noteworthy that, within a given complex or its counterpart, a T_1_ state activated by SOC interactions could result in longer wavelength absorption compared to that of an SOC-activated T_2_ state. Furthermore, a Ru/Os complex possessing lower lying triplet states could potentially yield states that are activated more effectively by SOC interactions. For the Ru complexes based on DX1, the ligands derived from *pdp* were found to influence the energy difference between the S_1_ state and the lower lying triplet states, albeit without significantly affecting the SOC matrix elements. A comparative analysis between DX-Ru-2Cl and DX-Ru-2Cl–P(OMe)_3_ revealed that the latter exhibited a more pronounced SOC-activated state, characterized by an oscillator strength double that of the former. This difference stemmed from the smaller S_1_–T_2_ energy difference in DX-Ru-2Cl–P(OMe)_3_ compared to DX-Ru-2Cl. On the other hand, DX-Ru-2Cl–N, featuring a *pdn* ligand containing nitrogen, exhibited lower lying T_1_ and T_2_ states compared to DX-Ru-2Cl and DX-Ru-2Cl–P(OMe)_3_, which had phosphorus-containing ligands. As a result, the T_1_ state in DX-Ru-2Cl–N was effectively activated by SOC, leading to an absorption wavelength longer than that observed for the DX-Ru-2Cl and DX-Ru-2Cl–P(OMe)_3_ complexes, where the longest wavelength absorptions through SOC were predominantly associated with their higher energy T_2_ states.

The presence of osmium (Os) atoms in the Os complexes has been observed to significantly enhance the SOC matrix elements, thereby favoring the activation of spin-forbidden triplet states compared to their counterparts in Ru complexes. Notably, the DX-Os-2Cl complex exhibited an SOC-activated state (ST_4_) within the NIR region, peaking at 1114 nm, with an associated oscillator strength of up to 0.027. This oscillator strength value is comparable to that of the spin-allowed singlet state in the Ru complex. For instance, the corresponding spin-allowed state (ST_7_) in DX-Ru-2Cl, with an absorption peak at 814 nm, exhibited an oscillator strength of 0.065. Due to the sizable SOC matrix elements inherent in the Os complexes, each DX-Os-2X complex (where X = Cl, Br, I, or NCS) manifested an additional weak transition denoted as ST_3_. This transition featured the longest wavelength absorption, activated through SOC interactions between the T_1_ state and the higher energy S_2_ state, despite a considerable energy gap between them. For instance, in the case of DX-Os-2Cl, the energy difference between the T_1_ and S_2_ states amounted to 0.63 eV, which is notably larger than the energy difference of 0.12 eV between the T_2_ and S_1_ states.

Our comprehensive investigation sheds light on the intricate interplay of ancillary ligands, electronic states, and SOC interactions in Ru/Os complexes, providing valuable insights for the design and optimization of materials with enhanced long-wavelength absorption properties, that hold great promise for various photovoltaic and optoelectronic applications.

## Supporting Information Available

Full spectra of all studied complexes (PDF)
