# Supplementary material for: Enhancing Near-Infrared Absorption in Terpyridyl Ru/Os Complexes with Ancillary Ligands to Activate Spin-Forbidden Transitions in Dye-Sensitized Solar Cells: A TDDFT Investigation
Source: J Phys Chem A. 2024 Jan 25;128(5):880–94. doi: 10.1021/acs.jpca.3c07554 (PMC10860138; doi:10.1021/acs.jpca.3c07554)
Supplement: Supplementary file 1 — jp3c07554_si_001.pdf [file jp3c07554_si_001.pdf]

SUPPORTING INFORMATION

Enhancing Near-Infrared Absorption in Terpyridyl Ru/Os Complexes with Ancillary Ligands to Activate Spin-Forbidden Transitions in Dye-Sensitized Solar Cells: A TDDFT Investigation

RatnaJuwita,† Jian-MingLiao,‡ Chia-YuanChen,*,‡,§ and Hui-Hsu Gavin Tsai*,‡,§

†Applied Science, Universitas Negeri Malang, 551312 Malang, Indonesia

‡Department of Chemistry, National Central University, No. 300, Zhongda Road, Zhongli District, Taoyuan City 32001, Taiwan

§Research Center of New Generation Light Driven Photovoltaic Module, National Central University, No. 300, Zhongda Road, Zhongli District, Taoyuan City 32001, Taiwan

*Email: chiayuan@ncu.edu.tw.

*Email: hhtsai@cc.ncu.edu.tw.

Table S1. Excitation wavelengths (nm), oscillator strengths (*f*), and contributions of SR states for DX-Ru-2Cl complex calculated with SR-TDDFT and SOC-TDDFT.

DX-Ru-2Cl			
Scalar Contributions (TDDFT Singlet-Singlet excitations)			
State	Wavelength (nm)	*f*	Compositions
S_1	822	0.073	(H→ L) (97%)
S_2	675	0.037	(H-1→ L) (80%), (H→ L+1) (18%)
S_3	564	0.018	(H-2→ L) (79%), (H-1→ L+1) (20%)
S_4	563	0.088	(H-1→ L+1) (78%), (H-2→ L) (20%)
S_5	539	0.067	(H→ L+1) (70%), (H-1→ L) (14%), (H→ L+3) (8%), (H-1→ L+2) (5%)
S_6	495	0.291	(H→ L+2) (98%)
S_7	463	0.138	(H-1→ L+2) (75%), (H→ L+3) (23%)
S_9	442	0.023	(H-1→ L+3) (78%), (H→ L+3) (15%)
S_{10}	439	0.085	(H→ L+3) (50%), (H-1→ L+3) (21%), (H-1→ L+2) (15%), (H→ L+1) (7%)
Scalar Contributions (TDDFT Singlet-Triplet excitations)			
State	Wavelength (nm)	f	Compositions
T_1	930	0.000	(H→ L) (93%), (H-1→ L) (5%)
T_2	894	0.000	(H-1→ L) (90%), (H→ L) (5%)
T_3	687	0.000	(H→ L+1) (94%)
T_4	600	0.000	(H-1→ L+1) (96%)
T_5	596	0.000	(H-2→ L) (96%)
T_6	518	0.000	(H→ L+2) (97%)
T_7	493	0.000	(H-1→ L+2) (93%)
T_8	486	0.000	(H→ L+3) (88%)

T_9	483	0.000	(H→ L+6) (83%), (H→ L+5) (8%)
T_{10}	469	0.000	(H-2→ L+1) (97%)
T_{11}	455	0.000	(H-1→ L+3) (97%)
T_{12}	437	0.000	(H-1→ L+6) (84%), (H-1→ L+5) (10%)
T_{13}	411	0.000	(H-4→ L) (78%), (H-5→ L) (9%)
T_{14}	404	0.000	(H-4→ L+1) (50%), (H-5→ L+1) (8%), (H-4→ L+3) (6%), (H-8→ L) (6%), (H-12→ L) (6%)
Spin Orbit Coupling Transitions			
State	Wavelength (nm)	f	Compositions
ST_3	936	0.0009	T_1 (48%), T_1 (48%), S_2 (1%), S_1 (1%)
ST_4	907	0.007	T_2 (44%), T_2 (44%), S_1 (10%)
ST_7	814	0.065	S_1 (87%), T_2 (5%), T_2 (5%)
ST_{11}	677	0.036	S_2 (96%), T_5 (2%)
ST_{19}	563	0.065	S_4 (68%), S_3 (31%)
ST_{24}	503	0.164	S_6 (56%), T_7 (21%), T_7 (21%)
ST_{31}	508	0.091	S_6 (31%), T_7 (24%), T_7 (24%), T_9 (7%), T_9 (7%), T_9 (7%)
ST_{37}	465	0.103	S_7 (74%), T_{11} (10%), T_{11} (10%)
ST_{44}	439	0.035	S_{10} (40%), T_{12} (23%), T_{12} (23%), T_{12} (6%)

Table S2. Excitation wavelengths (nm), oscillator strengths (*f*), and contributions of SR states for DX-Ru-2Cl-N complex calculated with SR-TDDFT and SOC-TDDFT.

DX-Ru-2Cl-N			
Scalar Contributions (TDDFT Singlet-Singlet excitations)			
State	Wavelength (nm)	*f*	Compositions
S_1	816	0.059	(H→ L) (96%)
S_2	665	0.030	(H-1→ L) (74%), (H→ L+1) (23%)
S_3	618	0.001	(H-2→ L) (95%)
S_4	572	0.137	(H-1→ L+1) (97%)
S_5	535	0.024	(H→ L+1) (61%), (H-1→ L) (17%), (H-1→ L+2) (12%), (H→ L+3) (7%)
S_6	496	0.315	(H→ L+2) (96%)
S_7	483	0.010	(H-2→ L+1) (89%), (H-1→ L+2) (9%)
S_8	466	0.199	(H-1→ L+2) (62%), (H→ L+3) (28%), (H-2→ L+1) (6%)
S_{10}	431	0.107	(H→ L+3) (60%), (H-1→ L+2) (15%), (H→ L+1) (10%), (H-1→ L) (5%)
Scalar Contributions (TDDFT Singlet-Triplet excitations)			
State	Wavelength (nm)	f	Compositions
T_1	1024	0.000	(H-1→ L) (91%)
T_2	946	0.000	(H→ L) (95%)
T_3	680	0.000	(H→ L+1) (57%), (H-2→ L) (38%)
T_4	676	0.000	(H-2→ L) (58%), (H→ L+1) (37%)
T_5	618	0.000	(H-1→ L+1) (98%)
T_6	522	0.000	(H→ L+2) (94%)
T_7	514	0.000	(H-1→ L+2) (91%), (H-1→ L) (5%)
T_8	509	0.000	(H→ L+6) (68%), (H→ L+5) (25%)

T_9	503	0.000	(H-2→ L+1) (95%)
T_{10}	481	0.000	(H→ L+3) (94%)
T_{11}	467	0.000	(H-1→ L+3) (72%), (H-1→ L+6) (17%), (H-1→ L+5) (7%)
T_{12}	465	0.000	(H-1→ L+6) (48%), (H-1→ L+3) (25%), (H-1→ L+5) (20%)
T_{13}	440	0.000	(H-2→ L+6) (66%), (H-2→ L+5) (27%)
T_{14}	417	0.000	(H-2→ L+2) (96)
T_{15}	404	0.000	(H-4→ L) (46%), (H-4→ L+1) (26%)
T_{16}	401	0.000	(H-4→ L) (38%), (H-4→ L+1) (25%), (H-6→ L) (5%)
Spin Orbit Coupling Transitions			
State	Wavelength (nm)	f	Compositions
ST_3	1036	0.002	T_1 (45%), T_1 (45%), T_1 (6%), S_1 (3%)
ST_4	953	0.0003	T_2 (46%), T_2 (46%), T_2 (6%), S_2 (2%)
ST_7	816	0.056	S_1 (95%), T_1 (3%)
ST_{14}	661	0.020	S_2 (66%), T_4 (17%), T_3 (8%), T_5 (4%), T_4 (2%)
ST_{19}	572	0.132	S_4 (96%), T_9 (2%), T_3 (2%)
ST_{33}	493	0.258	S_6 (82%), T_7 (14%)
ST_{44}	461	0.110	S_8 (55%), T_{11} (22%), T_{12} (20%)

Table S3. Excitation wavelengths (nm), oscillator strengths (*f*), and contributions of SR states for DX-Ru-2Cl-P(OMe)$_3$ complex calculated with SR-TDDFT and SOC-TDDFT.

DX-Ru-2Cl-P(OMe)$_3$			
Scalar Contributions (TDDFT Singlet-Singlet excitations)			
State	Wavelength (nm)	*f*	Compositions
S_1	813	0.076	(H→ L) (98%)
S_2	669	0.032	(H-1→ L) (82%), (H→ L+1) (17%)
S_3	552	0.087	(H-1→ L+1) (98%)
S_5	534	0.062	(H→ L+1) (70%), (H-1→ L) (13%), (H→ L+3) (7%), (H-1→ L+2) (4%), (H-2→ L) (3%)
S_6	490	0.285	(H→ L+2) (98%)
S_7	457	0.104	(H-1→ L+2) (71%), (H→ L+3) (28%)
S_9	437	0.062	(H→ L+3) (54%), (H-1→ L+2) (19%), (H-1→ L+3) (14%), (H→ L+1) (6%)
S_{10}	433	0.022	(H-1→ L+3) (86%), (H→ L+3) (8%), (H-1→ L+2) (4%)
Scalar Contributions (TDDFT Singlet-Triplet excitations)			
State	Wavelength (nm)	f	Compositions
T_1	914	0.000	(H→ L) (95%), (H-1→ L) (3%)
T_2	848	0.000	(H-1→ L) (92%), (H→ L) (3%)
T_3	679	0.000	(H→ L+1) (94%)
T_5	570	0.000	(H-2→ L) (98%)
T_6	513	0.000	(H→ L+2) (97%)
T_7	482	0.000	(H→ L+3) (86%), (H-1→ L+2) (9%)
T_9	480	0.000	(H-1→ L+2) (85%), (H→ L+3) (9%)
T_{10}	454	0.000	(H-2→ L+1) (97%)
Spin Orbit Coupling Transitions			

State	Wavelength (nm)	f	Compositions
ST_3	920	0.0006	T_1 (98%), S_2 (1%)
ST_4	866	0.017	T_2 (77%), S_1 (22%)
ST_7	801	0.059	S_1 (77%), T_2 (22%)
ST_{11}	670	0.031	S_2 (97%), T_1 (1%)
ST_{18}	552	0.085	S_3 (97%), T_3 (1%)
ST_{24}	496	0.21	S_6 (74%), T_9 (20%), T_7 (4%), T_8 (1%)

Table S4. Excitation wavelengths (nm), oscillator strengths (*f*), and contributions of SR states for DX-Ru-2Br complex calculated with SR-TDDFT and SOC-TDDFT.

DX-Ru-2Br			
Scalar Contributions (TDDFT Singlet-Singlet excitations)			
State	Wavelength (nm)	*f*	Compositions
S_1	802	0.064	(H→ L) (96%)
S_2	679	0.037	(H-1→ L) (85%), (H→ L+1) (12%)
S_3	569	0.001	(H-2→ L) (96%)
S_4	564	0.093	(H-1→ L+1) (95%)
S_5	545	0.060	(H→ L+1) (77%), (H-1→ L) (11%)
S_6	492	0.253	(H→ L+2) (98%)
S_7	465	0.115	(H-1→ L+2) (82%), (H→ L+3) (8%), (H-1→ L) (7%)
S_8	459	0.014	(H-2→ L+1) (85%), (H→ L+3) (8%)
S_9	446	0.020	(H→ L+3) (35%), (H→ L+6) (29%), (H→ L+5) (21%), (H-1→ L+2) (5%)
S_{10}	444	0.021	(H→ L+3) (38%), (H→ L+6) (24%), (H→ L+5) (17%), (H-1→ L+3) (6%)
S_{12}	430	0.009	(H-3→ L) (97%)
Scalar Contributions (TDDFT Singlet-Triplet excitations)			

State	Wavelength (nm)	f	Compositions
T_1	902	0.000	(H→ L) (87%), (H-1→ L) (9%)
T_2	854	0.000	(H-1→ L) (86%), (H→ L) (10%)
T_3	673	0.000	(H→ L+1) (93%)
T_4	600	0.000	(H-2→ L) (94%)
T_5	594	0.000	(H-1→ L+1) (96%)
T_6	523	0.000	(H→ L+6) (53%), (H→ L+5) (39%)
T_7	513	0.000	(H→ L+2) (95%)
T_8	491	0.000	(H-1→ L+2) (94%)
T_9	481	0.000	(H→ L+3) (93%)
T_{10}	476	0.000	(H-2→ L+1) (73%), (H-1→ L+6) (12%), (H-1→ L+5) (10%)
T_{11}	472	0.000	(H-1→ L+6) (38%), (H-1→ L+5) (31%), (H-2→ L+1) (22%)
T_{12}	453	0.000	(H-1→ L+3) (96%)
T_{13}	436	0.000	(H-3→ L) (93%)
T_{14}	422	0.000	(H-5→ L) (72%), (H-4→ L) (7%)
T_{15}	414	0.000	(H-2→ L+6) (48%), (H-2→ L+5) (37%)
T_{16}	413	0.000	(H-5→ L+1) (29%), (H-6→ L) (23%), (H-9→ L+1) (8%), (H-12→ L) (6%)
T_{17}	402	0.000	(H-4→ L) (78%), (H-5→ L) (8%), (H-2→ L+2) (8%)
Spin Orbit Coupling Transitions			
State	Wavelength (nm)	f	Compositions
ST_3	915	0.003	T_1 (46%), T_1 (46%), S_1 (4%)
ST_4	877	0.012	T_2 (39%), T_2 (39%), S_1 (18%)
ST_7	785	0.050	S_1 (78%), T_2 (10%), T_2 (10%)
ST_{11}	677	0.024	S_2 (62%), T_3 (16%), T_3 (16%)
ST_{19}	561	0.088	S_4 (94%), T_3 (2%), T_3 (2%)

ST_{20}	544	0.047	S_5 (78%), T_6 (10%), T_5 (4%), T_5 (4%)
ST_{27}	506	0.129	S_6 (51%), T_8 (24%), T_8 (24%)
ST_{34}	478	0.114	S_6 (45%), T_8 (23%), T_8 (23%), T_{10} (3%)
ST_{40}	466	0.054	S_7 (44%), T_{12} (14%), T_{12} (14%), S_8 (6%), S_{10} (5%)

Table S5. Excitation wavelengths (nm), oscillator strengths (*f*), and contributions of SR states for DX-Ru-2I complex calculated with SR-TDDFT and SOC-TDDFT.

DX-Ru-2I			
Scalar Contributions (TDDFT Singlet-Singlet excitations)			
State	Wavelength (nm)	*f*	Compositions
S_1	798	0.056	(H→ L) (97%)
S_2	705	0.030	(H-1→ L) (92%), (H→ L+1) (6%)
S_3	582	0.002	(H-2→ L) (89%), (H-4→ L) (6%)
S_4	577	0.065	(H-1→ L+1) (98%)
S_5	565	0.045	(H→ L+1) (88%), (H-1→ L) (5%)
S_7	497	0.184	(H→ L+2) (97%)
S_9	478	0.074	(H-1→ L+2) (92%)
S_{14}	439	0.047	(H-5→ L) (63%), (H-6→ L) (28%), (H-1→ L+4) (5%)
S_{17}	421	0.080	(H-6→ L) (64%), (H-5→ L) (28%)
Scalar Contributions (TDDFT Singlet-Triplet excitations)			
State	Wavelength (nm)	f	Compositions
T_1	881	0.000	(H→ L) (86%), (H-1→ L) (9%)
T_2	837	0.000	(H-1→ L) (85%), (H→ L) (10%)
T_3	669	0.000	(H→ L+1) (91%)

T_4	610	0.000	(H-2→ L) (85%), (H-4→ L) (7%)
T_5	601	0.000	(H-1→ L+1) (97%)
T_6	561	0.000	(H→ L+4) (80%), (H→ L+6) (5%)
T_7	526	0.000	(H-3→ L) (66%), (H-1→ L+4) (25%)
T_8	514	0.000	(H→ L+2) (94%)
T_9	500	0.000	(H-1→ L+4) (42%), (H-3→ L) (23%), (H-1→ L+2) (21%)
T_{10}	498	0.000	(H-1→ L+2) (64%), (H-1→ L+4) (17%), (H-3→ L) (5%)
T_{11}	486	0.000	(H-2→ L+1) (85%), (H-4→ L+1) (7%)
T_{12}	484	0.000	(H→ L+3) (84%), (H-5→ L) (6%)
T_{13}	471	0.000	(H-4→ L) (62%), (H-6→ L) (15%), (H-5→ L) (8%)
T_{14}	469	0.000	(H-6→ L) (32%), (H-5→ L) (25%), (H-4→ L) (25%), (H-1→ L+3) (6%)
T_{15}	495	0.000	(H-1→ L+3) (71%), (H-5→ L) (20%)
T_{16}	458	0.000	(H-6→ L) (38%), (H-5→ L) (23%), (H-1→ L+3) (16%), (H→ L+3) (7%), (H-1→ L+2) (6%), (H-7→ L) (5%)
T_{17}	448	0.000	(H-2→ L+4) (61%), (H-4→ L+4) (23%)
T_{18}	428	0.000	(H-7→ L) (21%), (H-6→ L+1) (20%), (H-3→ L+1) (11%), (H-5→ L+1) (10%), (H-8→ L+1) (7%), (H-5→ L) (6%)
T_{19}	426	0.000	(H-3→ L+1) (84%)
T_{20}	405	0.000	(H-2→ L+2) (84%), (H-7→ L) (6%)
Spin Orbit Coupling Transitions			
State	Wavelength (nm)	f	Compositions
ST_3	921	0.010	T_1 (35%), T_1 (35%), S_1 (15%), T_2 (4%), T_2 (4%)
ST_4	895	0.013	T_2 (29%), T_2 (29%), S_1 (19%), T_1 (8%), T_1 (8%)
ST_7	757	0.036	S_1 (64%), T_2 (15%), T_2 (15%)
ST_{12}	628	0.012	T_5 (28%), T_5 (28%), S_5 (25%), T_4 (7%), T_4 (7%), T_4 (2%)
ST_{21}	567	0.043	S_4 (66%), T_6 (10%), T_6 (10%), T_3 (4%), T_3 (4%), T_7 (2%)

ST_{23}	548	0.024	S_5 (51%), T_5 (10%), T_5 (10%), T_7 (9%), T_7 (9%), T_{13} (2%), T_8 (2%), T_8 (2%)
ST_{31}	526	0.071	S_7 (37%), T_{10} (14%), T_{10} (14%), T_9 (8%), T_9 (8%), T_8 (5%), T_8 (5%), S_9 (5%)
ST_{49}	472	0.075	S_7 (40%), T_{10} (10%), T_{10} (10%), T_9 (8%), T_9 (8%), T_{10} (4%), S_8 (3%), T_{14} (2%), T_{15} (2%), T_{15} (2%)

Table S6. Excitation wavelengths (nm), oscillator strengths (*f*), and contributions of SR states for DX-Ru-2NCS complex calculated with SR-TDDFT and SOC-TDDFT.

DX-Ru-2NCS			
Scalar Contributions (TDDFT Singlet-Singlet excitations)			
State	Wavelength (nm)	*f*	Compositions
S_1	878	0.050	(H→ L) (98%)
S_2	755	0.069	(H-1→ L) (91%), (H→ L+1) (8%)
S_3	614	0.084	(H-1→ L+1) (97%)
S_4	601	0.064	(H→ L+1) (87%), (H-1→ L) (7%)
S_6	518	0.167	(H→ L+2) (98%)
S_7	502	0.074	(H-3→ L) (97%)
S_8	497	0.131	(H-1→ L+2) (94%)
S_9	476	0.011	(H→ L+3) (93%)
Scalar Contributions (TDDFT Singlet-Triplet excitations)			
State	Wavelength (nm)	f	Compositions
T_1	956	0.000	(H→ L) (85%), (H-1→ L) (11%)
T_2	946	0.000	(H-1→ L) (84%), (H→ L) (11%)
T_3	695	0.000	(H→ L+1) (95%)
T_4	643	0.000	(H-1→ L+1) (98%)
T_5	576	0.000	(H-2→ L) (76%), (H-4→ L) (22%)

T_6	534	0.000	(H→ L+2) (97%)
T_7	524	0.000	(H-1→ L+2) (93%)
T_8	512	0.000	(H-3→ L) (95%)
T_9	500	0.000	(H→ L+3) (96%)
T_{10}	483	0.000	(H-1→ L+3) (97%)
T_{11}	470	0.000	(H-4→ L) (75%), (H-2→ L) (22%)
T_{12}	461	0.000	(H-2→ L+1) (81%), (H-4→ L+1) (16%)
T_{13}	440	0.000	(H-5→ L) (81%), (H-7→ L) (8%)
T_{14}	426	0.000	(H-6→ L) (32%), (H-5→ L+1) (22%), (H-7→ L+1) (17%), (H-11→ L) (6%)
T_{15}	422	0.000	(H-3→ L+1) (97%)
Spin Orbit Coupling Transitions			
State	Wavelength (nm)	f	Compositions
ST_3	962	0.002	T_1 (45%), T_1 (45%), S_1 (3%), T_2 (3%), T_2 (3%)
ST_4	954	0.003	T_2 (43%), T_2 (43%), S_1 (6%), T_1 (4%), T_1 (4%)
ST_7	871	0.046	S_1 (91%), T_2 (4%), T_2 (4%)
ST_8	754	0.068	S_2 (98%), T_1 (1%)
ST_{15}	613	0.082	S_3 (97%), T_3 (2%)
ST_{16}	599	0.061	S_4 (95%), T_4 (2%), T_4 (2%)
ST_{24}	529	0.055	T_7 (33%), T_7 (33%), S_6 (33%)
ST_{27}	514	0.102	S_6 (61%), T_7 (16%), T_7 (16%), T_8 (4%), T_8 (4%)
ST_{33}	502	0.073	S_7 (98%), T_9 (1%)
ST_{35}	496	0.124	S_8 (94%), T_6 (2%), T_6 (2%), T_{10} (1%)

Table S7. Excitation wavelengths (nm), oscillator strengths (*f*), and contributions of SR states for BD-Ru-3Cl complex calculated with SR-TDDFT and SOC-TDDFT.

BD-Ru-3Cl			
Scalar Contributions (TDDFT Singlet-Singlet excitations)			
State	Wavelength (nm)	*f*	Compositions
S_1	834	0.071	(H-1→ L) (92%)
S_2	796	0.001	(H-2→ L) (96%)
S_3	695	0.033	(H→ L) (77%), (H-1→ L+1) (20%)
S_4	602	0.172	(H→ L+1) (95%)
S_7	517	0.381	(H-1→ L+1) (93%)
S_9	484	0.289	(H→ L+2) (49%), (H-1→ L+3) (34%), (H-1→ L+1) (12%)
S_{12}	433	0.106	(H-1→ L+3) (58%), (H-1→ L+1) (17%), (H→ L+2) (12%), (H→ L) (7%)
S_{13}	407	0.031	(H-3→ L) (92%), (H-1→ L+5) (6%)
Scalar Contributions (TDDFT Singlet-Triplet excitations)			
State	Wavelength (nm)	f	Compositions
T_1	1255	0.000	(H→ L) (88%), (H→ L+2) (7%)
T_2	1000	0.000	(H-1→ L) (94%)
T_3	907	0.000	(H-2→ L) (95%)
T_4	705	0.000	(H-1→ L+1) (92%)
T_5	664	0.000	(H→ L+1) (95%)
T_6	615	0.000	(H-2→ L+1) (97%)
T_7	563	0.000	(H→ L+2) (89%), (H→ L) (8%)
T_8	549	0.000	(H-1→ L+2) (94%)
T_9	505	0.000	(H-2→ L+2) (96%)

T_{10}	495	0.000	(H→ L+3) (95%)
T_{11}	493	0.000	(H-1→ L+3) (94%)
T_{12}	480	0.000	(H-1→ L+5) (93%)
T_{13}	475	0.000	(H-2→ L+5) (94%)
T_{14}	454	0.000	(H→ L+5) (87%), (H-2→ L+3) (9%)
T_{15}	451	0.000	(H-2→ L+3) (89%), (H→ L+5) (9%)
T_{16}	419	0.000	(H-3→ L) (95%)
T_{17}	398	0.000	(H-4→ L+1) (43%), (H→ L+4) (26%), (H-4→ L+3) (10%), (H-10→ L) (5%)
T_{18}	395	0.000	(H-4→ L) (88%), (H-10→ L+1) (5%)
T_{19}	375	0.000	(H-1→ L+4) (66%), (H→ L+4) (24%)
T_{20}	374	0.000	(H→ L+4) (43%), (H-1→ L+4) (30%), (H-4→ L+1) (14%)
Spin Orbit Coupling Transitions			
State	Wavelength (nm)	f	Compositions
ST_7	910	0.005	T_3 (89%), S_1 (8%)
ST_{10}	826	0.065	S_1 (91%), T_3 (8%)
ST_{15}	698	0.028	S_3 (84%), T_5 (12%), T_6 (2%)
ST_{22}	595	0.131	S_4 (76%), T_6 (22%)
ST_{31}	518	0.296	S_7 (78%), T_9 (14%), T_7 (8%)
ST_{45}	481	0.224	S_9 (77%), T_{10} (12%), T_9 (3%), T_{12} (3%), T_{11} (2%)
ST_{57}	431	0.096	S_{12} (91%), T_{15} (6%), T_{12} (2%)
ST_{78}	362	0.064	S_{18} (88%), T_{19} (10%)

Table S8. Excitation wavelengths (nm), oscillator strengths (*f*), and contributions of SR states for BD-Ru-3Br complex calculated with SR-TDDFT and SOC-TDDFT.

BD-Ru-3Br			
Scalar Contributions (TDDFT Singlet-Singlet excitations)			
State	Wavelength (nm)	*f*	Compositions
S_1	820	0.067	(H→ L) (94%)
S_2	793	0.001	(H-2→ L) (96%)
S_3	691	0.042	(H-1→ L) (81%), (H→ L+1) (17%)
S_4	592	0.143	(H-1→ L+1) (96%)
S_7	513	0.336	(H→ L+2) (95%)
S_{10}	484	0.318	(H-1→ L+2) (59%), (H→ L+3) (20%), (H→ L+1) (10%), (H-3→ L) (7%)
S_{14}	442	0.064	(H→ L+3) (71%), (H→ L+1) (10%), (H-1→ L+2) (8%)
S_{16}	419	0.018	(H-4→ L) (95%)
Scalar Contributions (TDDFT Singlet-Triplet excitations)			
State	Wavelength (nm)	f	Compositions
T_1	1139	0.000	(H-1→ L) (89%), (H-1→ L+2) (6%)
T_2	960	0.000	(H→ L) (95%)
T_3	888	0.000	(H-2→ L) (95%)
T_4	690	0.000	(H→ L+1) (93%)
T_5	646	0.000	(H-1→ L+1) (97%)
T_6	613	0.000	(H-2→ L+1) (97%)
T_7	549	0.000	(H-1→ L+2) (89%), (H-1→ L) (7%)
T_8	542	0.000	(H→ L+2) (95%)
T_9	530	0.000	(H→ L+5) (93%)

T_{10}	526	0.000	(H-2→ L+5) (90%)
T_{11}	507	0.000	(H-3→ L) (98%)
T_{12}	505	0.000	(H-2→ L+2) (96%)
T_{13}	502	0.000	(H-1→ L+5) (96%)
T_{14}	489	0.000	(H→ L+3) (94%)
T_{15}	485	0.000	(H-1→ L+3) (96%)
T_{16}	453	0.000	(H-2→ L+3) (98%)
T_{17}	426	0.000	(H-4→ L) (98%)
T_{18}	419	0.000	(H-3→ L+1) (97%)
T_{19}	416	0.000	(H-5→ L) (98%)
T_{20}	412	0.000	(H-6→ L) (86%)
Spin Orbit Coupling Transitions			
State	Wavelength (nm)	f	Compositions
ST_7	887	0.003	T_3 (90%), S_1 (5%)
ST_{10}	812	0.062	S_1 (92%), T_3 (6%)
ST_{15}	693	0.036	S_3 (87%), T_5 (8%), T_6 (2%)
ST_{22}	585	0.116	S_4 (81%), T_6 (15%), T_4 (2%)
ST_{37}	511	0.172	S_7 (51%), T_{12} (20%), T_7 (14%), T_{11} (14%), T_{13} (2%)
ST_{53}	478	0.222	S_{10} (70%), T_{15} (20%), T_{12} (3%), S_9 (2%), T_{13} (2%), T_8 (2%)
ST_{62}	440	0.054	S_{14} (83%), T_{16} (8%), T_{17} (4%), T_{15} (2%), T_{18} (2%)
ST_{80}	381	0.021	S_{20} (99%)

Table S9. Excitation wavelengths (nm), oscillator strengths (*f*), and contributions of SR states for BD-Ru-3I complex calculated with SR-TDDFT and SOC-TDDFT.

BD-Ru-3I			
Scalar Contributions (TDDFT Singlet-Singlet excitations)			
State	Wavelength (nm)	*f*	Compositions
S_1	815	0.056	(H→ L) (95%)
S_2	795	0.001	(H-2→ L) (94%)
S_3	696	0.045	(H-1→ L) (85%), (H→ L+1) (12%)
S_4	633	0.012	(H-3→ L) (99%)
S_5	591	0.007	(H-2→ L+1) (87%), (H-1→ L+1) (9%)
S_6	588	0.101	(H-1→ L+1) (87%), (H-2→ L+1) (9%)
S_7	555	0.018	(H→ L+1) (74%), (H-1→ L+2) (11%), (H-1→ L) (10%)
S_8	517	0.254	(H→ L+2) (94%)
S_9	516	0.012	(H-4→ L) (98%)
S_{13}	491	0.229	(H-1→ L+2) (82%), (H→ L+3) (6%), (H→ L+1) (6%)
S_{15}	475	0.020	(H-1→ L+4) (93%)
S_{17}	459	0.022	(H-3→ L+2) (82%), (H→ L+3) (17%)
S_{18}	455	0.011	(H→ L+3) (40%), (H-2→ L+3) (38%), (H-3→ L+2) (11%)
S_{20}	453	0.022	(H-2→ L+4) (42%), (H-1→ L+3) (30%), (H-7→ L) (16%)
Scalar Contributions (TDDFT Singlet-Triplet excitations)			
State	Wavelength (nm)	f	Compositions
T_1	1046	0.000	(H-1→ L) (89%), (H-1→ L+2) (5%)
T_2	933	0.000	(H→ L) (94%)
T_3	874	0.000	(H-2→ L) (92%)

T_4	684	0.000	(H→ L+1) (91%)
T_5	648	0.000	(H-3→ L) (98%)
T_6	633	0.000	(H-1→ L+1) (96%)
T_7	617	0.000	(H-2→ L+1) (94%)
T_8	584	0.000	(H→ L+4) (90%)
T_9	579	0.000	(H-2→ L+4) (83%), (H-8→ L+4) (10%)
T_{10}	552	0.000	(H-1→ L+4) (90%)
T_{11}	541	0.000	(H→ L+2) (95%)
T_{12}	540	0.000	(H-1→ L+2) (88%), (H-1→ L) (6%)
T_{13}	524	0.000	(H-4→ L) (95%)
T_{14}	513	0.000	(H-3→ L+1) (99%)
T_{15}	512	0.000	(H-2→ L+2) (83%), (H-5→ L) (10%)
T_{16}	499	0.000	(H-5→ L) (86%), (H-2→ L+2) (11%)
T_{17}	490	0.000	(H→ L+3) (91%)
T_{18}	479	0.000	(H-1→ L+3) (87%), (H-7→ L) (7%)
T_{19}	466	0.000	(H-7→ L) (70%), (H-6→ L) (14%), (H-1→ L+3) (10%)
T_{20}	462	0.000	(H-3→ L+2) (88%), (H-6→ L) (6%)
Spin Orbit Coupling Transitions			
State	Wavelength (nm)	f	Compositions
ST_3	1091	0.004	T_1 (86%), S_1 (6%)
ST_4	966	0.002	T_2 (82%), T_3 (12%), S_3 (5%)
ST_{10}	815	0.056	S_1 (87%), T_1 (5%)
ST_{15}	698	0.038	S_3 (84%), T_6 (6%), T_2 (4%)
ST_{31}	574	0.071	S_6 (70%), T_8 (12%), T_7 (8%), S_5 (4%), T_4 (4%)
ST_{49}	519	0.079	S_8 (31%), T_{15} (21%), T_{15} (21%), T_{20} (18%), T_{16} (6%)

ST_{61}	485	0.084	S_{13} (37%), T_{16} (35%), T_{13} (12%), T_{11} (6%), T_{14} (2%)

Table S10. Excitation wavelengths (nm), oscillator strengths (*f*), and contributions of SR states for BD-Ru-3NCS complex calculated with SR-TDDFT and SOC-TDDFT.

BD-Ru-3NCS			
Scalar Contributions (TDDFT Singlet-Singlet excitations)			
State	Wavelength (nm)	*f*	Compositions
S_1	872	0.051	(H-1→ L) (97%)
S_2	784	0.001	(H-2→ L) (95%)
S_3	771	0.093	(H→ L) (89%), (H-1→ L+1) (9%)
S_4	641	0.125	(H→ L+1) (98%)
S_5	593	0.022	(H-1→ L+1) (81%), (H→ L+2) (8%), (H→ L) (7%)
S_7	533	0.205	(H-1→ L+2) (98%)
S_8	526	0.099	(H→ L+2) (69%), (H-3→ L) (24%)
S_9	518	0.243	(H-3→ L) (76%), (H→ L+2) (20%)
S_{13}	488	0.077	(H-5→ L) (100%)
S_{14}	474	0.011	(H-1→ L+3) (94%)
S_{16}	437	0.004	(H-3→ L+1) (100%)
S_{17}	415	0.020	(H-4→ L+1) (100%)
S_{19}	409	0.013	(H-6→ L) (97%)
Scalar Contributions (TDDFT Singlet-Triplet excitations)			
State	Wavelength (nm)	f	Compositions
T_1	1134	0.000	(H→ L) (93%)

T_2	975	0.000	(H-1→ L) (95%)
T_3	846	0.000	(H-2→ L) (94%)
T_4	697	0.000	(H-1→ L+1) (94%)
T_5	688	0.000	(H→ L+1) (97%)
T_6	602	0.000	(H-2→ L+1) (96%)
T_7	574	0.000	(H→ L+2) (93%)
T_8	552	0.000	(H-1→ L+2) (96%)
T_9	532	0.000	(H-3→ L) (99%)
T_{10}	509	0.000	(H→ L+3) (96%)
T_{11}	501	0.000	(H-1→ L+3) (96%)
T_{12}	498	0.000	(H-2→ L+2) (74%), (H-4→ L) (22%)
T_{13}	497	0.000	(H-4→ L) (76%), (H-2→ L+2) (22%)
T_{14}	495	0.000	(H-5→ L) (99%)
T_{15}	451	0.000	(H-2→ L+3) (97%)
T_{16}	438	0.000	(H-6→ L) (47%), (H-3→ L+1) (45%)
T_{17}	437	0.000	(H-3→ L+1) (55%), (H-6→ L) (40%)
T_{18}	420	0.000	(H-6→ L+1) (26%), (H-8→ L) (25%), (H-9→ L+1) (19%), (H-9→ L+3) (5%), (H-10→ L) (5%)
T_{19}	417	0.000	(H-4→ L+1) (99%)
T_{20}	414	0.000	(H-5→ L+1) (100%)
Spin Orbit Coupling Transitions			
State	Wavelength (nm)	f	Compositions
ST_4	983	0.001	T_2 (95%), T_3 (3%), S_3 (2%)
ST_7	878	0.040	S_1 (80%), T_3 (19%)
ST_{12}	768	0.089	S_3 (96%), T_3 (2%)

ST_{19}	640	0.113	S_4 (91%), T_4 (6%), T_6 (3%)
ST_{23}	590	0.017	S_5 (77%), T_6 (11%), T_6 (11%)
ST_{34}	532	0.174	S_7 (85%), T_9 (10%), T_7 (4%)
ST_{36}	518	0.229	S_9 (94%), T_{10} (4%)
ST_{39}	510	0.011	T_{10} (90%), S_9 (4%), S_{14} (3%)
ST_{74}	417	0.013	S_{17} (65%), T_{20} (35%)

Table S11. Excitation wavelengths (nm), oscillator strengths (*f*), and contributions of SR states for DX-Os-2Cl complex calculated with SR-TDDFT and SOC-TDDFT.

DX-Os-2Cl			
Scalar Contributions (TDDFT Singlet-Singlet excitations)			
State	Wavelength (nm)	*f*	Compositions
S_1	929	0.105	(H→ L) (97%)
S_2	716	0.032	(H-1→ L) (77%), (H→ L+1) (22%)
S_3	596	0.001	(H-2→ L) (98%)
S_4	577	0.091	(H-1→ L+1) (96%)
S_5	561	0.061	(H→ L+1) (63%), (H-1→ L) (15%), (H→ L+3) (12%), (H-1→ L+2) (9%)
S_6	536	0.482	(H→ L+2) (95%)
S_7	484	0.173	(H-1→ L+2) (69%), (H→ L+3) (30%)
S_8	463	0.002	(H-2→ L+1) (94%)
S_9	453	0.014	(H-1→ L+3) (97%)
S_{10}	447	0.206	(H→ L+3) (53%), (H-1→ L+2) (20%), (H→ L+1) (13%), (H-1→ L) (6%)
S_{11}	403	0.002	(H-2→ L+2) (99%)
Scalar Contributions (TDDFT Singlet-Triplet excitations)			
State	Wavelength (nm)	f	Compositions

T_1	1125	0.000	(H→ L) (97%)
T_2	1024	0.000	(H-1→ L) (92%)
T_3	768	0.000	(H→ L+1) (91%)
T_4	640	0.000	(H-2→ L) (97%)
T_5	628	0.000	(H-1→ L+1) (98%)
T_6	576	0.000	(H→ L+2) (97%)
T_7	530	0.000	(H-1→ L+2) (71%), (H→ L+3) (24%)
T_8	522	0.000	(H→ L+3) (71%), (H-1→ L+2) (23%)
T_9	485	0.000	(H-2→ L+1) (96%)
T_{10}	471	0.000	(H-1→ L+3) (97%)
T_{11}	407	0.000	(H-3→ L) (60%), (H-4→ L) (17%), (H-2→ L+2) (8%), (H-5→ L) (5%)
T_{12}	406	0.000	(H-2→ L+2) (90%), (H-3→ L) (5%)
T_{13}	400	0.000	(H→ L+7) (85%), (H→ L+5) (10%)
T_{14}	397	0.000	(H-3→ L+1) (42%), (H-4→ L+1) (12%), (H-1→ L+4) (7%), (H-3→ L+3) (5%), (H-11→ L) (5%), (H-5→ L+1) (5%)
T_{15}	391	0.000	(H→ L+4) (95%)
T_{16}	377	0.000	(H-4→ L) (60%), (H-3→ L) (16%), (H-1→ L+7) (10%), (H-6→ L) (9%)
T_{17}	376	0.000	(H-2→ L+3) (95%)
T_{18}	365	0.000	(H-1→ L+4) (78%), (H-8→ L) (6%)
T_{19}	362	0.000	(H-1→ L+7) (70%), (H-1→ L+5) (15%), (H-4→ L) (8%)
T_{20}	352	0.000	(H→ L+5) (86%), (H→ L+7) (10%)
Spin Orbit Coupling Transitions			
State	Wavelength (nm)	f	Compositions
ST_3	1180	0.002	T_1 (46%), T_1 (46%), S_2 (4%)
ST_4	1114	0.027	T_2 (35%), T_2 (35%), S_1 (26%)

ST_7	900	0.070	S_1 (67%), T_2 (14%), T_2 (14%)
ST_{11}	729	0.025	S_2 (79%), T_5 (5%), T_5 (5%), T_4 (4%), T_1 (3%), T_1 (3%)
ST_{22}	576	0.088	S_4 (85%), T_3 (3%), T_3 (3%), S_6 (2%), T_9 (2%)
ST_{23}	562	0.260	S_6 (53%), T_7 (17%), T_7 (17%), S_4 (5%), T_8 (2%), T_8 (2%)
ST_{30}	510	0.195	S_6 (40%), T_7 (18%), T_7 (18%), T_8 (11%), T_8 (11%)
ST_{40}	445	0.155	S_{10} (75%), T_{10} (8%), T_{10} (8%), T_{13} (4%)
ST_{67}	362	0.256	S_{15} (79%), T_{18} (7%), T_{18} (7%), S_{12} (5%)
ST_{70}	360	0.057	T_{18} (27%), T_{18} (27%), S_{15} (18%), S_{12} (16%), T_{19} (10%)
ST_{77}	350	0.053	S_{17} (83%), T_{15} (3%), T_{15} (3%), S_{16} (3%), T_{19} (3%), T_{19} (3%)

Table S12. Excitation wavelengths (nm), oscillator strengths (*f*), and contributions of SR states for DX-Os-2Br complex calculated with SR-TDDFT and SOC-TDDFT.

DX-Os-2Br			
Scalar Contributions (TDDFT Singlet-Singlet excitations)			
State	Wavelength (nm)	*f*	Compositions
S_1	903	0.097	(H→ L) (97%)
S_2	717	0.034	(H-1→ L) (83%), (H→ L+1) (16%)
S_3	607	0.001	(H-2→ L) (98%)
S_4	575	0.085	(H-1→ L+1) (96%)
S_5	566	0.065	(H→ L+1) (71%), (H-1→ L) (12%), (H→ L+3) (8%), (H-1→ L+2) (6%)
S_6	531	0.427	(H→ L+2) (96%)
S_7	485	0.173	(H-1→ L+2) (78%), (H→ L+3) (18%)
S_8	472	0.003	(H-2→ L+1) (90%), (H→ L+3) (7%)
S_9	455	0.112	(H→ L+3) (64%), (H-1→ L+2) (14%), (H→ L+1) (9%), (H-2→ L+1) (6%)

S_{10}	452	0.007	(H-1→ L+3) (99%)
S_{11}	411	0.022	(H-3→ L) (98%)
S_{12}	410	0.005	(H-2→ L+2) (98%)
Scalar Contributions (TDDFT Singlet-Triplet excitations)			
State	Wavelength (nm)	f	Compositions
T_1	1073	0.000	(H→ L) (95%)
T_2	966	0.000	(H-1→ L) (91%)
T_3	748	0.000	(H→ L+1) (90%)
T_4	649	0.000	(H-2→ L) (97%)
T_5	620	0.000	(H-1→ L+1) (98%)
T_6	568	0.000	(H→ L+2) (96%)
T_7	525	0.000	(H-1→ L+2) (83%), (H→ L+3) (11%)
T_8	516	0.000	(H→ L+3) (82%), (H-1→ L+2) (11%)
T_9	494	0.000	(H-2→ L+1) (95%)
T_{10}	469	0.000	(H-1→ L+3) (97%)
T_{11}	442	0.000	(H→ L+6) (83%), (H→ L+5) (11%)
T_{12}	424	0.000	(H-3→ L) (87%), (H-2→ L+6) (8%)
T_{13}	416	0.000	(H-5→ L) (63%), (H-4→ L) (23%)
T_{14}	414	0.000	(H-2→ L+2) (95%)
T_{15}	403	0.000	(H-5→ L+1) (30%), (H-6→ L) (16%), (H-4→ L+1) (10%), (H-8→ L+1) (6%), (H-12→ L) (6%)
T_{16}	399	0.000	(H-1→ L+6) (74%), (H-1→ L+5) (12%), (H-3→ L) (9%)
T_{17}	388	0.000	(H-4→ L) (63%), (H-5→ L) (26%)
T_{18}	387	0.000	(H→ L+4) (94%)

T_{19}	382	0.000	(H-2→ L+3) (95%)
T_{20}	379	0.000	(H-6→ L) (46%), (H-1→ L+4) (18%), (H-9→ L) (13%), (H-5→ L+1) (8%)
Spin Orbit Coupling Transitions			
State	Wavelength (nm)	f	Compositions
ST_3	1133	0.003	T_1 (44%), T_1 (44%), S_2 (5%)
ST_4	1066	0.030	T_2 (31%), T_2 (31%), S_1 (31%)
ST_7	860	0.058	S_1 (60%), T_2 (17%), T_2 (17%)
ST_{11}	725	0.024	S_2 (77%), T_4 (4%), T_5 (4%), T_5 (4%), T_1 (3%), T_1 (3%)
ST_{22}	572	0.083	S_4 (80%), T_3 (4%), T_3 (4%), S_6 (4%), T_7 (2%), T_7 (2%), T_9 (2%)
ST_{23}	561	0.222	S_6 (51%), T_7 (18%), T_7 (18%), S_4 (7%)
ST_{30}	502	0.179	S_6 (42%), T_7 (22%), T_7 (22%), T_8 (5%), T_8 (5%)
ST_{31}	499	0.059	S_7 (23%), T_{10} (16%), T_{10} (16%), S_9 (14%), T_9 (9%), T_9 (9%), S_8 (3%), T_{11} (2%)
ST_{35}	473	0.084	S_7 (40%), S_8 (23%), S_9 (12%), T_{10} (5%), T_{10} (5%), T_{11} (4%), T_9 (2%), T_9 (2%), T_6 (2%), T_6 (2%),
ST_{43}	441	0.046	S_9 (38%), T_{11} (26%), T_{12} (5%), T_{12} (5%), T_{10} (5%), T_{10} (5%), T_{16} (4%), T_{16} (4%), S_7 (2%)
ST_{80}	347	0.096	S_{20} (89%), S_{19} (3%), T_{18} (3%), T_{18} (3%)

Table S13. Excitation wavelengths (nm), oscillator strengths (*f*), and contributions of SR states for DX-Os-2I complex calculated with SR-TDDFT and SOC-TDDFT.

DX-Os-2I			
Scalar Contributions (TDDFT Singlet-Singlet excitations)			
State	Wavelength (nm)	*f*	Compositions
S_1	891	0.091	(H→ L) (98%)
S_2	739	0.031	(H-1→ L) (90%), (H→ L+1) (9%)
S_3	616	0.001	(H-2→ L) (96%)
S_4	584	0.068	(H-1→ L+1) (97%)
S_5	581	0.058	(H→ L+1) (83%), (H-1→ L) (7%)
S_6	533	0.338	(H→ L+2) (97%)
S_7	495	0.132	(H-1→ L+2) (89%), (H→ L+3) (5%)
S_8	493	0.026	(H-3→ L) (94%)
S_9	480	0.003	(H-2→ L+1) (94%)
S_{10}	469	0.015	(H→ L+3) (85%)
S_{11}	459	0.003	(H-4→ L) (90%)
S_{12}	458	0.005	(H-1→ L+3) (95%)
S_{13}	433	0.043	(H-5→ L) (98%)
S_{14}	426	0.001	(H→ L+5) (70%), (H→ L+6) (22%)
S_{15}	417	0.001	(H-2→ L+2) (97%)
S_{16}	406	0.079	(H-6→ L) (90%)
S_{17}	405	0.004	(H-3→ L+1) (95%)
Scalar Contributions (TDDFT Singlet-Triplet excitations)			
State	Wavelength (nm)	f	Compositions

T_1	1028	0.000	(H→ L) (96%)
T_2	931	0.000	(H-1→ L) (92%)
T_3	736	0.000	(H→ L+1) (90%)
T_4	652	0.000	(H-2→ L) (95%)
T_5	620	0.000	(H-1→ L+1) (97%)
T_6	565	0.000	(H→ L+2) (97%)
T_7	527	0.000	(H-1→ L+2) (90%)
T_8	517	0.000	(H→ L+3) (89%)
T_9	508	0.000	(H-3→ L) (90%)
T_{10}	501	0.000	(H-2→ L+1) (91%)
T_{11}	489	0.000	(H→ L+5) (62%), (H→ L+6) (22%), (H-4→ L) (12%)
T_{12}	473	0.000	(H-1→ L+3) (83%), (H-5→ L) (12%)
T_{13}	464	0.000	(H-4→ L) (50%), (H-5→ L) (30%), (H-1→ L+3) (8%), (H→ L+5) (5%)
T_{14}	462	0.000	(H-5→ L) (48%), (H-4→ L) (32%), (H-1→ L+3) (7%)
T_{15}	445	0.000	(H-6→ L) (74%), (H-7→ L) (7%)
T_{16}	440	0.000	(H-1→ L+5) (68%), (H-1→ L+6) (22%)
T_{17}	420	0.000	(H-2→ L+2) (97%)
T_{18}	415	0.000	(H-5→ L+1) (34%), (H-7→ L) (20%), (H-6→ L) (12%), (H-8→ L+1) (9%)
T_{19}	406	0.000	(H-3→ L+1) (98%)
T_{20}	396	0.000	(H-7→ L) (51%), (H-5→ L+1) (14%), (H-8→ L) (10%)
Spin Orbit Coupling Transitions			
State	Wavelength (nm)	f	Compositions
ST_3	1109	0.005	T_1 (42%), T_1 (42%), S_2 (10%)
ST_4	1069	0.035	T_2 (27%), T_2 (27%), S_1 (38%)
ST_7	821	0.044	S_1 (47%), T_2 (19%), T_2 (19%), T_3 (6%), T_3 (6%)

ST_{11}	733	0.024	S_2 (74%), T_1 (6%), T_1 (6%), T_5 (4%), T_5 (4%), T_4 (2%)
ST_{22}	578	0.127	S_6 (32%), S_4 (30%), T_7 (14%), T_7 (14%), T_3 (3%), T_3 (3%)
ST_{23}	573	0.105	S_4 (50%), S_6 (21%), T_7 (8%), T_7 (8%), T_3 (4%), T_3 (4%)
ST_{31}	525	0.020	S_8 (65%), T_{13} (5%), T_{13} (5%), T_{14} (4%), T_{14} (4%), T_{11} (3%), T_{11} (3%), S_7 (2%)
ST_{38}	496	0.079	S_6 (23%), T_{10} (15%), T_7 (12%), T_7 (12%), T_{11} (5%), T_{11} (5%), T_{15} (4%), T_{15} (4%), T_9 (3%), T_{16} (2%), S_{13} (2%), T_8 (2%), T_8 (2%)
ST_{39}	495	0.062	T_{10} (20%), S_6 (18%), T_7 (12%), T_7 (12%), T_{11} (11%), T_{11} (11%), T_{11} (4%), T_{16} (4%), T_9 (2%)
ST_{65}	414	0.010	S_{15} (25%), S_{13} (23%), T_{15} (9%), T_{15} (9%), T_{18} (8%), T_{18} (8%), T_{17} (5%), T_{17} (2%), T_{17} (2%)
ST_{75}	385	0.040	S_{16} (50%), T_{13} (7%), T_{14} (5%), T_{20} (5%), T_{20} (5%), S_{18} (5%), T_9 (5%), T_9 (5%), S_{19} (3%), S_{17} (2%), T_{11} (2%), T_{18} (2%), T_{18} (2%)

Table S14. Excitation wavelengths (nm), oscillator strengths (*f*), and contributions of SR states for DX-Os-2NCS complex calculated with SR-TDDFT and SOC-TDDFT.

DX-Os-2NCS			
Scalar Contributions (TDDFT Singlet-Singlet excitations)			
State	Wavelength (nm)	*f*	Compositions
S_1	1020	0.075	(H→ L) (98%)
S_2	815	0.060	(H-1→ L) (86%), (H→ L+1) (13%)
S_3	641	0.102	(H-1→ L+1) (98%)
S_4	632	0.087	(H→ L+1) (81%), (H-1→ L) (11%)
S_5	582	0.002	(H-2→ L) (88%), (H-4→ L) (11%)
S_6	566	0.297	(H→ L+2) (98%)
S_7	524	0.198	(H-1→ L+2) (91), (H→ L+3) (6%)
S_8	501	0.042	(H→ L+3) (88%), (H-1→ L+2) (5%)
S_9	489	0.013	(H-1→ L+3) (99%)

S_{10}	488	0.135	(H-3→ L) (98%)
S_{11}	459	0.002	(H-4→ L) (78%), (H-2→ L) (10%), (H-2→ L+1) (10%)
S_{12}	457	0.028	(H-2→ L+1) (79%), (H-4→ L) (9%), (H-4→ L+1) (9%)
S_{13}	406	0.002	(H-3→ L+1) (99%)
S_{14}	404	0.001	(H-5→ L) (98%)
Scalar Contributions (TDDFT Singlet-Triplet excitations)			
State	Wavelength (nm)	f	Compositions
T_1	1180	0.000	(H→ L) (97%)
T_2	1109	0.000	(H-1→ L) (94%)
T_3	786	0.000	(H→ L+1) (94%)
T_4	688	0.000	(H-1→ L+1) (98%)
T_5	614	0.000	(H-2→ L) (83%), (H-4→ L) (16%)
T_6	597	0.000	(H→ L+2) (97%)
T_7	569	0.000	(H-1→ L+2) (93%)
T_8	541	0.000	(H→ L+3) (94%)
T_9	506	0.000	(H-1→ L+3) (97%)
T_{10}	503	0.000	(H-3→ L) (98%)
T_{11}	473	0.000	(H-2→ L+1) (85%), (H-4→ L+1) (13%)
T_{12}	467	0.000	(H-4→ L) (82%), (H-2→ L) (16%)
T_{13}	436	0.000	(H-5→ L) (85%), (H-7→ L) (6%)
T_{14}	418	0.000	(H-6→ L) (31%), (H-5→ L+1) (24%), (H-7→ L+1) (16%), (H-11→ L) (5%)
T_{15}	407	0.000	(H-3→ L+1) (99%)
T_{16}	403	0.000	(H→ L+4) (97%)
T_{17}	399	0.000	(H-2→ L+2) (93%), (H-4→ L+2) (5%)

T_{18}	396	0.000	(H-6→ L) (43%), (H-1→ L+4) (34%), (H-5→ L+1) (9%)
T_{19}	387	0.000	(H-7→ L) (71%), (H-8→ L) (13%), (H-5→ L) (8%)
T_{20}	385	0.000	(H-4→ L+1) (70%), (H-2→ L+1) (12%), (H-1→ L+4) (10%)
Spin Orbit Coupling Transitions			
State	Wavelength (nm)	f	Compositions
ST_3	1214	0.003	T_1 (47%), T_1 (47%), S_2 (3%)
ST_4	1177	0.019	T_2 (36%), T_2 (36%), S_1 (25%)
ST_7	986	0.055	S_1 (73%), T_2 (13%), T_2 (13%)
ST_8	815	0.055	S_2 (92%), T_1 (2%), T_1 (2%), T_4 (2%), T_4 (2%)
ST_{12}	697	0.013	T_4 (41%), T_4 (41%), S_4 (13%), S_2 (3%)
ST_{15}	636	0.095	S_3 (93%), T_3 (3%), T_3 (3%)
ST_{16}	629	0.069	S_4 (78%), T_4 (6%), T_4 (6%), T_6 (3%), T_6 (3%)
ST_{23}	590	0.140	S_6 (47%), T_7 (26%), T_7 (26%)
ST_{27}	551	0.120	S_6 (40%), T_7 (20%), T_7 (20%), T_8 (9%), T_8 (9%), S_9 (2%)
ST_{31}	525	0.132	S_7 (65%), T_9 (11%), T_9 (11%), S_8 (9%), T_6 (2%), T_6 (2%)
ST_{39}	488	0.115	S_{10} (83%), S_8 (7%), T_9 (4%), T_9 (4%)
ST_{48}	455	0.027	S_{12} (95%), S_{11} (3%), T_4 (1%)
ST_{77}	379	0.030	S_{17} (98%), T_{19} (1%)

Table S15. Excitation wavelengths (nm), oscillator strengths (*f*), and contributions of SR states for BD-Os-3Cl complex calculated with SR-TDDFT and SOC-TDDFT.

BD-Os-3Cl			
Scalar Contributions (TDDFT Singlet-Singlet excitations)			
State	Wavelength (nm)	*f*	Compositions
S_1	848	0.031	(H-2→ L) (67%), (H→ L) (26%), (H-2→ L+2) (3%)
S_2	845	0.081	(H→ L) (62%), (H-2→ L) (28%), (H→ L+2) (5%)
S_3	693	0.010	(H-1→ L) (67%), (H→ L+1) (30%)
S_5	594	0.156	(H-1→ L+1) (88%), (H→ L+2) (9%)
S_6	556	0.025	(H-1→ L+2) (43%), (H→ L+1) (33%), (H-1→ L) (12%), (H→ L+3) (9%)
S_7	540	0.116	(H-2→ L+2) (79%), (H→ L+2) (13%), (H-2→ L) (4%)
S_8	538	0.630	(H→ L+2) (67%), (H-2→ L+2) (15%), (H→ L) (8%), (H-1→ L+1) (5%)
S_9	482	0.256	(H→ L+3) (64%), (H-1→ L+2) (28%), (H→ L+1) (3%)
Scalar Contributions (TDDFT Singlet-Triplet excitations)			
State	Wavelength (nm)	f	Compositions
T_1	1098	0.000	(H-1→ L) (82%), (H-1→ L+2) (11%)
T_2	1053	0.000	(H→ L) (90%), (H→ L+2) (4%), (H-1→ L) (2%)
T_3	1017	0.000	(H-2→ L) (90%), (H-2→ L+2) (8%)
T_4	730	0.000	(H-1→ L+1) (93%), (H-1→ L+3) (2%)
T_5	674	0.000	(H-1→ L+1) (96%)
T_6	660	0.000	(H-2→ L+1) (97%), (H-2→ L+3) (2%)
T_7	604	0.000	(H-1→ L+2) (81%), (H-1→ L) (12%), (H→ L+2) (4%)
T_8	594	0.000	(H→ L+2) (89%), (H-1→ L+2) (4%), (H→ L) (3%)
T_9	548	0.000	(H-2→ L+2) (91%), (H-2→ L) (8%)

T_{10}	516	0.000	(H-1→ L+3) (95%), (H-1→ L+1) (2%)
T_{11}	507	0.000	(H-1→ L+3) (97%)
T_{12}	473	0.000	(H-2→ L+3) (97%), (H-2→ L+1) (2%)
Spin Orbit Coupling Transitions			
State	Wavelength (nm)	f	Compositions
ST_2	1255	0.005	T_1 (52%), T_2 (35%), S_1 (7%)
ST_3	1232	0.006	T_1 (57%), T_3 (33%), S_2 (6%)
ST_7	983	0.003	T_3 (54%), T_1 (40%), S_2 (3%)
ST_{10}	827	0.032	S_1 (73%), S_2 (11%), T_3 (8%)
ST_{11}	825	0.064	S_2 (73%), S_1 (11%), T_2 (5%), T_1 (2%), T_3 (3%)
ST_{27}	581	0.132	S_5 (83%), T_6 (8%), T_7 (3%), T_4 (2%)
ST_{30}	550	0.117	S_8 (17%), S_6 (17%), T_9 (31%), T_7 (20%), T_8 (6%), S_7 (3%)
ST_{31}	549	0.063	S_6 (50%), S_8 (7%), T_8 (11%), T_9 (8%), T_7 (7%), T_{11} (5%), T_9 (2%)
ST_{37}	526	0.162	S_7 (72%), S_8 (13%), T_8 (10%), T_7 (3%)
ST_{39}	518	0.326	S_8 (50%), T_9 (40%), S_7 (8%)
ST_{42}	478	0.123	S_9 (48%), T_{11} (34%), T_{12} (13%)

Table S16. Excitation wavelengths (nm), oscillator strengths (*f*), and contributions of SR states for BD-Os-3Br complex calculated with SR-TDDFT and SOC-TDDFT.

BD-Os-3Br			
Scalar Contributions (TDDFT Singlet-Singlet excitations)			
State	Wavelength (nm)	*f*	Compositions
S_1	868	0.012	(H-2→ L) (82%), (H→ L) (13%)
S_2	856	0.093	(H→ L) (78%), (H-2→ L) (14%)
S_3	708	0.026	(H-1→ L) (80%), (H→ L+1) (19%)
S_4	619	0.001	(H-2→ L+1) (98%)
S_5	597	0.164	(H-1→ L+1) (95%)
S_7	546	0.453	(H→ L+2) (79%), (H-2→ L+2) (11%), (H→ L) (6%)
S_8	543	0.076	(H-2→ L+2) (84%), (H→ L+2) (10%)
S_9	495	0.343	(H-1→ L+2) (52%), (H→ L+3) (36%), (H→ L+1) (9%)
S_{12}	461	0.014	(H-3→ L) (97%)
S_{13}	442	0.198	(H→ L+3) (55%), (H→ L+1) (16%), (H-1→ L+2) (16%), (H-1→ L) (6%)
Scalar Contributions (TDDFT Singlet-Triplet excitations)			
State	Wavelength (nm)	f	Compositions
T_1	1222	0.000	(H-1→ L) (84%), (H-1→ L+2) (11%)
T_2	1073	0.000	(H→ L) (93%)
T_3	1015	0.000	(H-2→ L) (91%), (H-2→ L+2) (6%)
T_4	745	0.000	(H→ L+1) (91%)
T_5	671	0.000	(H-1→ L+1) (97%)
T_6	662	0.000	(H-2→ L+1) (96%)
T_7	596	0.000	(H-1→ L+2) (84%), (H-1→ L) (13%)

T_8	593	0.000	(H→ L+2) (94%)
T_9	551	0.000	(H-2→ L+2) (93%), (H-2→ L) (6%)
T_{10}	517	0.000	(H→ L+3) (94%)
T_{11}	503	0.000	(H-1→ L+3) (96%)
T_{12}	477	0.000	(H-2→ L+3) (97%)
T_{13}	476	0.000	(H-3→ L) (98%)
T_{14}	451	0.000	(H-2→ L+5) (95%)
T_{15}	416	0.000	(H→ L+5) (96%)
T_{16}	400	0.000	(H-1→ L+5) (95%)
T_{17}	397	0.000	(H-1→ L+4) (49%), (H-6→ L+1) (27%), (H-7→ L+3) (5%)
T_{18}	396	0.000	(H-4→ L) (93%)
T_{19}	393	0.000	(H-6→ L) (69%), (H-3→ L+1) (15%), (H-5→ L) (6%)
T_{20}	412	0.000	(H-3→ L+1) (81%), (H-5→ L) (10%), (H-6→ L) (8%)
Spin Orbit Coupling Transitions			
State	Wavelength (nm)	f	Compositions
ST_2	1453	0.002	T_1 (61%), T_2 (12%), T_2 (12%), S_1 (8%), T_1 (3%)
ST_3	1412	0.007	T_1 (34%), T_1 (34%), T_3 (11%), T_3 (11%), S_2 (6%), T_1 (2%)
ST_4	1239	0.002	T_3 (33%), T_2 (28%), T_2 (28%), S_3 (5%), T_2 (3%), T_3 (2%)
ST_7	982	0.004	T_3 (31%), T_3 (31%), T_1 (13%), T_1 (13%), S_2 (4%)
ST_{10}	843	0.012	S_1 (80%), T_2 (5%)
ST_{11}	837	0.070	S_2 (74%), S_1 (4%), T_3 (4%), T_3 (4%), T_4 (3%), T_4 (3%), T_2 (3%)
ST_{27}	582	0.139	S_5 (84%), T_6 (9%), T_4 (4%)
ST_{42}	482	0.250	S_9 (72%), T_{11} (20%), S_{13} (3%), T_9 (2%)
ST_{56}	445	0.136	S_{13} (67%), T_{15} (8%), T_{14} (5%), T_{14} (5%), T_{12} (5%), T_{16} (5%)

Table S17. Excitation wavelengths (nm), oscillator strengths (*f*), and contributions of SR states for BD-Os-3I complex calculated with SR-TDDFT and SOC-TDDFT.

BD-Os-3I			
Scalar Contributions (TDDFT Singlet-Singlet excitations)			
State	Wavelength (nm)	*f*	Compositions
S_1	882	0.001	(H-1→ L) (93%)
S_2	852	0.101	(H→ L) (91%)
S_3	713	0.036	(H-2→ L) (84%), (H→ L+1) (15%)
S_4	630	0.001	(H-1→ L+1) (97%)
S_5	594	0.130	(H-2→ L+1) (95%)
S_6	592	0.012	(H-3→ L) (98%)
S_9	546	0.440	(H→ L+2) (89%), (H→ L) (5%)
S_{10}	500	0.359	(H-2→ L+2) (69%), (H→ L+3) (19%), (H→ L+1) (9%)
S_{11}	481	0.025	(H-4→ L) (82%), (H-1→ L+3) (14%)
S_{16}	459	0.056	(H→ L+3) (72%), (H-2→ L+2) (8%), (H→ L+1) (8%)
S_{17}	443	0.054	(H-3→ L+2) (96%)
S_{20}	418	0.057	(H-6→ L (88%), (H-1→ L+5) (9%)
Scalar Contributions (TDDFT Singlet-Triplet excitations)			
State	Wavelength (nm)	f	Compositions
T_1	1134	0.000	(H-2→ L) (85%), (H-2→ L+2) (9%)
T_2	1037	0.000	(H→ L) (93%)
T_3	1011	0.000	(H-1→ L) (90%)
T_4	734	0.000	(H→ L+1) (90%)
T_5	670	0.000	(H-1→ L+1) (95%)

T_6	658	0.000	(H-2→ L+1) (97%)
T_7	608	0.000	(H-3→ L) (97%)
T_8	588	0.000	(H→ L+2) (95%)
T_9	583	0.000	(H-2→ L+2) (84%), (H-2→ L) (10%)
T_{10}	559	0.000	(H-1→ L+2) (92%), (H-1→ L) (5%)
T_{11}	516	0.000	(H→ L+3) (93%)
T_{12}	511	0.000	(H-1→ L+5) (91%), (H-8→ L+5) (5%)
T_{13}	506	0.000	(H→ L+5) (91%), (H-5→ L) (5%)
T_{14}	495	0.000	(H-2→ L+3) (94%)
T_{15}	492	0.000	(H-4→ L) (93%), (H-2→ L+5) (5%)
T_{16}	485	0.000	(H-1→ L+3) (95%)
T_{17}	481	0.000	(H-3→ L+1) (99%)
T_{18}	472	0.000	(H-5→ L) (92%), (H→ L+5) (5%)
T_{19}	468	0.000	(H-2→ L+5) (92%), (H-4→ L) (5%)
T_{20}	447	0.000	(H-3→ L+2) (91%), (H-6→ L) (6%)
Spin Orbit Coupling Transitions			
State	Wavelength (nm)	f	Compositions
ST_3	1350	0.010	T_1 (30%), T_1 (30%), T_3 (14%), T_3 (14%), S_2 (10%)
ST_4	1216	0.003	T_3 (36%), T_2 (24%), T_2 (24%), S_3 (7%), T_2 (3%), T_3 (3%)
ST_{11}	841	0.077	S_2 (75%), T_4 (8%), T_3 (5%)
ST_{31}	578	0.102	S_5 (77%), T_5 (6%), T_4 (6%), T_{19} (3%)
ST_{52}	493	0.176	S_{10} (48%), T_{17} (16%), S_{16} (7%), T_{16} (7%), T_{19} (6%), T_8 (4%), T_{18} (3%), T_{10} (2%), S_7 (2%), T_{15} (2%)

Table S18. Excitation wavelengths (nm), oscillator strengths (*f*), and contributions of SR states for BD-Os-3NCS complex calculated with SR-TDDFT and SOC-TDDFT.

BD-Os-3NCS			
Scalar Contributions (TDDFT Singlet-Singlet excitations)			
State	Wavelength (nm)	*f*	Compositions
S_1	942	0.079	(H-1→ L) (89%), (H→ L) (7%)
S_2	869	0.001	(H-2→ L) (97%)
S_3	806	0.085	(H→ L) (81%), (H-1→ L+1) (11%), (H-1→ L) (6%)
S_4	661	0.172	(H→ L+1) (90%), (H-1→ L+1) (6%)
S_5	625	0.001	(H-2→ L+1) (93%)
S_6	617	0.015	(H-1→ L+1) (63%), (H→ L+2) (14%), (H→ L) (8%), (H-2→ L+1) (5%), (H→ L+1) (5%)
S_7	571	0.349	(H-1→ L+2) (89%), (H→ L+2) (7%)
S_8	540	0.427	(H→ L+2) (75%), (H-1→ L+1) (10%), (H-1→ L+2) (6%), (H-1→ L+3) (5%)
S_{11}	491	0.012	(H-3→ L+3) (50%), (H-1→ L) (43%)
S_{12}	489	0.145	(H-3→ L) (50%), (H-1→ L+3) (41%)
S_{15}	457	0.156	(H-5→ L) (99%)
Scalar Contributions (TDDFT Singlet-Triplet excitations)			
State	Wavelength (nm)	f	Compositions
T_1	1271	0.000	(H→ L) (85%), (H-1→ L) (6%)
T_2	1118	0.000	(H-1→ L) (89%), (H→ L) (7%)
T_3	972	0.000	(H-2→ L) (94%)
T_4	766	0.000	(H-1→ L+1) (87%), (H→ L+1) (6%)
T_5	733	0.000	(H→ L+1) (90%), (H-1→ L+1) (7%)
T_6	653	0.000	(H-2→ L+1) (96%)

T_7	629	0.000	(H→ L+2) (85%), (H-1→ L+2) (7%), (H→ L) (5%)
T_8	607	0.000	(H-1→ L+2) (89%), (H→ L+2) (7%)
T_9	541	0.000	(H-2→ L+2) (96%)
T_{10}	532	0.000	(H→ L+3) (81%), (H-1→ L+3) (15%)
T_{11}	531	0.000	(H-1→ L+3) (81%), (H→ L+3) (15%)
T_{12}	509	0.000	(H-3→ L) (98%)
T_{13}	474	0.000	(H-2→ L+3) (98%)
T_{14}	471	0.000	(H-4→ L) (97%)
T_{15}	469	0.000	(H-5→ L) (97%)
T_{16}	424	0.000	(H-6→ L) (88%)
T_{17}	416	0.000	(H→ L+4) (48%), (H-6→ L+1) (18%), (H-9→ L+1) (11%), (H-9→ L+3) (6%)
T_{18}	415	0.000	(H-3→ L+1) (99%)
T_{19}	398	0.000	(H→ L+4) (37%), (H-8→ L) (26%), (H-6→ L+1) (13%), (H-9→ L+1) (7%)
T_{20}	395	0.000	(H-4→ L+1) (98%)
Spin Orbit Coupling Transitions			
State	Wavelength (nm)	f	Compositions
ST_3	1362	0.006	T_1 (81%), S_1 (7%), T_3 (12%)
ST_4	1202	0.004	T_2 (75%), T_3 (20%), S_3 (5%)
ST_7	985	0.026	T_3 (48%), S_1 (33%), T_1 (18%)
ST_{10}	892	0.048	S_1 (60%), T_3 (39%)
ST_{14}	805	0.064	S_3 (75%), T_5 (16%), T_3 (3%), T_6 (3%), T_2 (2%)
ST_{15}	799	0.017	T_4 (78%), S_4 (10%), T_6 (10%)
ST_{19}	661	0.092	S_4 (54%), T_6 (26%), T_4 (20%)
ST_{31}	570	0.252	S_7 (72%), T_7 (17%), T_9 (10%)
ST_{34}	552	0.150	S_8 (35%), T_{10} (33%), T_9 (12%), T_8 (9%), T_{13} (4%), S_{11} (2%), S_{12} (2%)
ST_{40}	524	0.208	S_8 (49%), T_9 (45%), T_{10} (5%)

ST_{48}	489	0.120	S_{12} (83%), T_{10} (8%), T_{13} (8%)
ST_{60}	456	0.149	S_{15} (95%), T_{14} (5%)

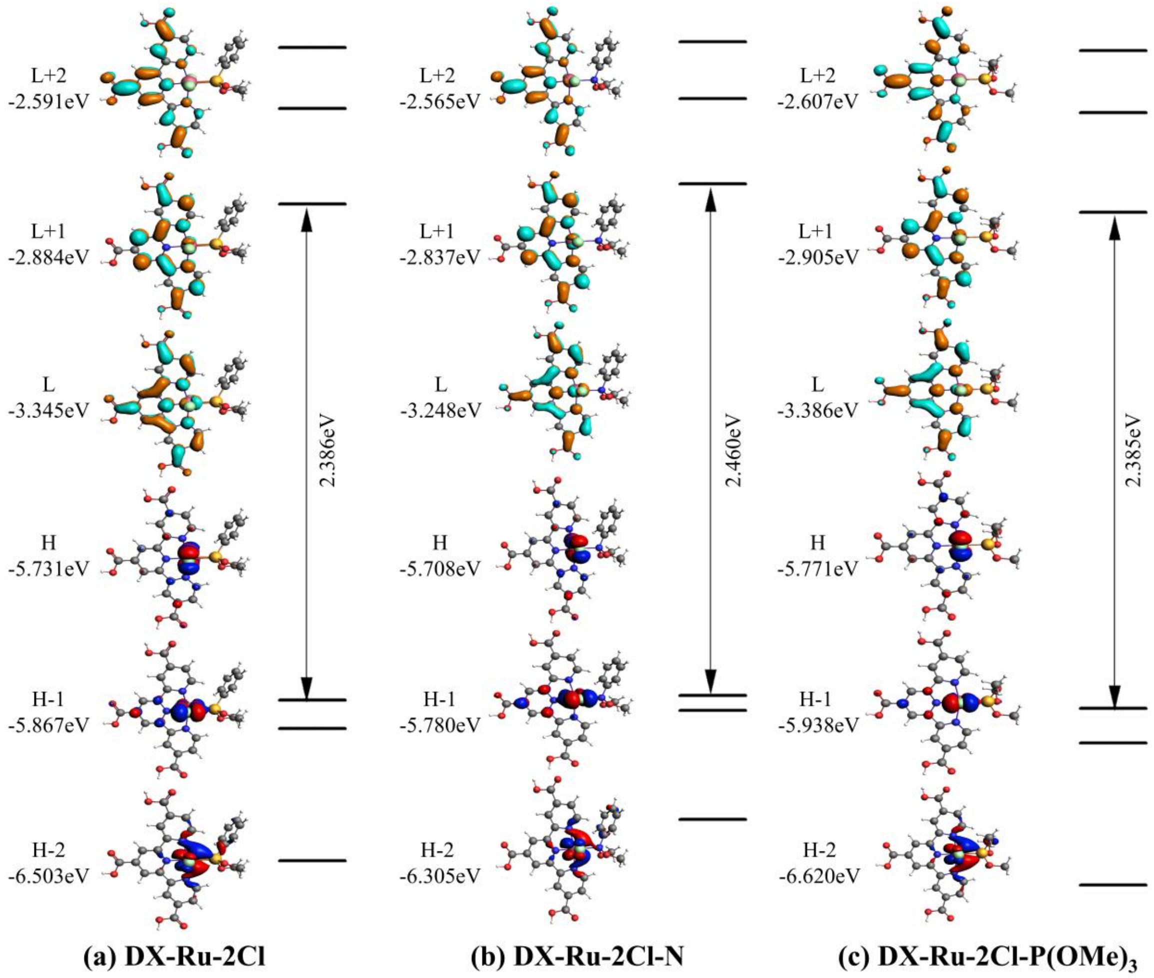

Figure S1. Six frontier molecular orbitals of (a) DX-Ru-2Cl, (b) DX-Ru-2Cl-N, and (c) DX-Ru-2Cl-P(OMe)$_3$ calculated by SR-TDDFT. Blue, gray, green, orange, red, white and pink spheres represent N, C, Cl, P, O, H and Ru atoms, respectively.

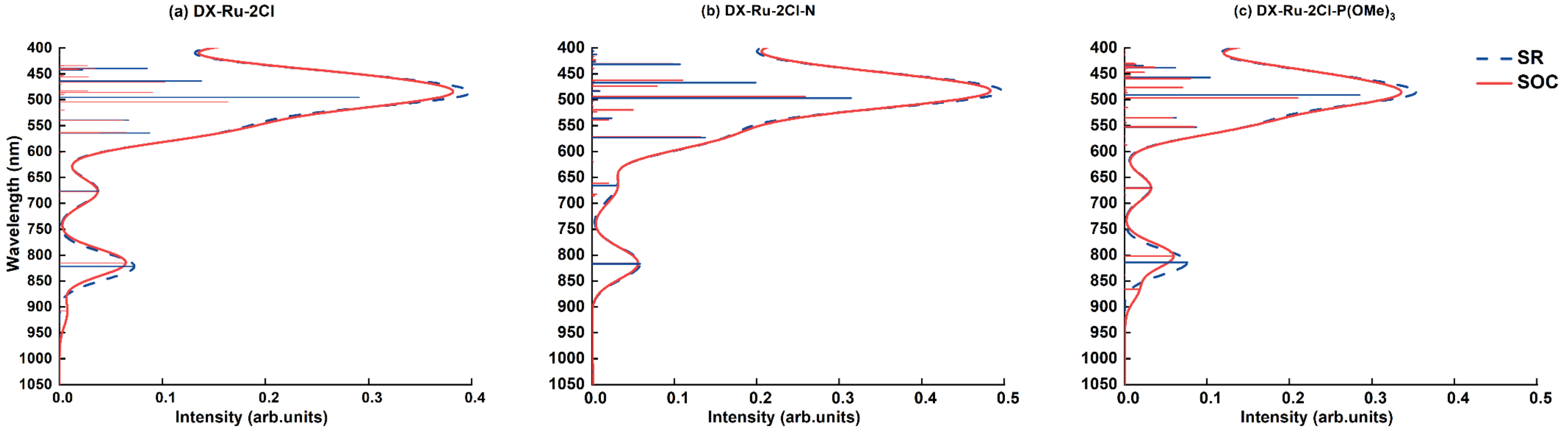

Figure S2. Simulated absorption spectra (λ > 400 nm) of (a) DX-Ru-2Cl, (b) DX-Ru-2Cl-N, and (c) DX-Ru-2Cl-P(OMe)$_3$ complexes in DMF. Excitation energies and spectra calculated by SR-TDDFT are shown in blue and excitation energies and spectra calculated by SO-TDDFT are shown in red.

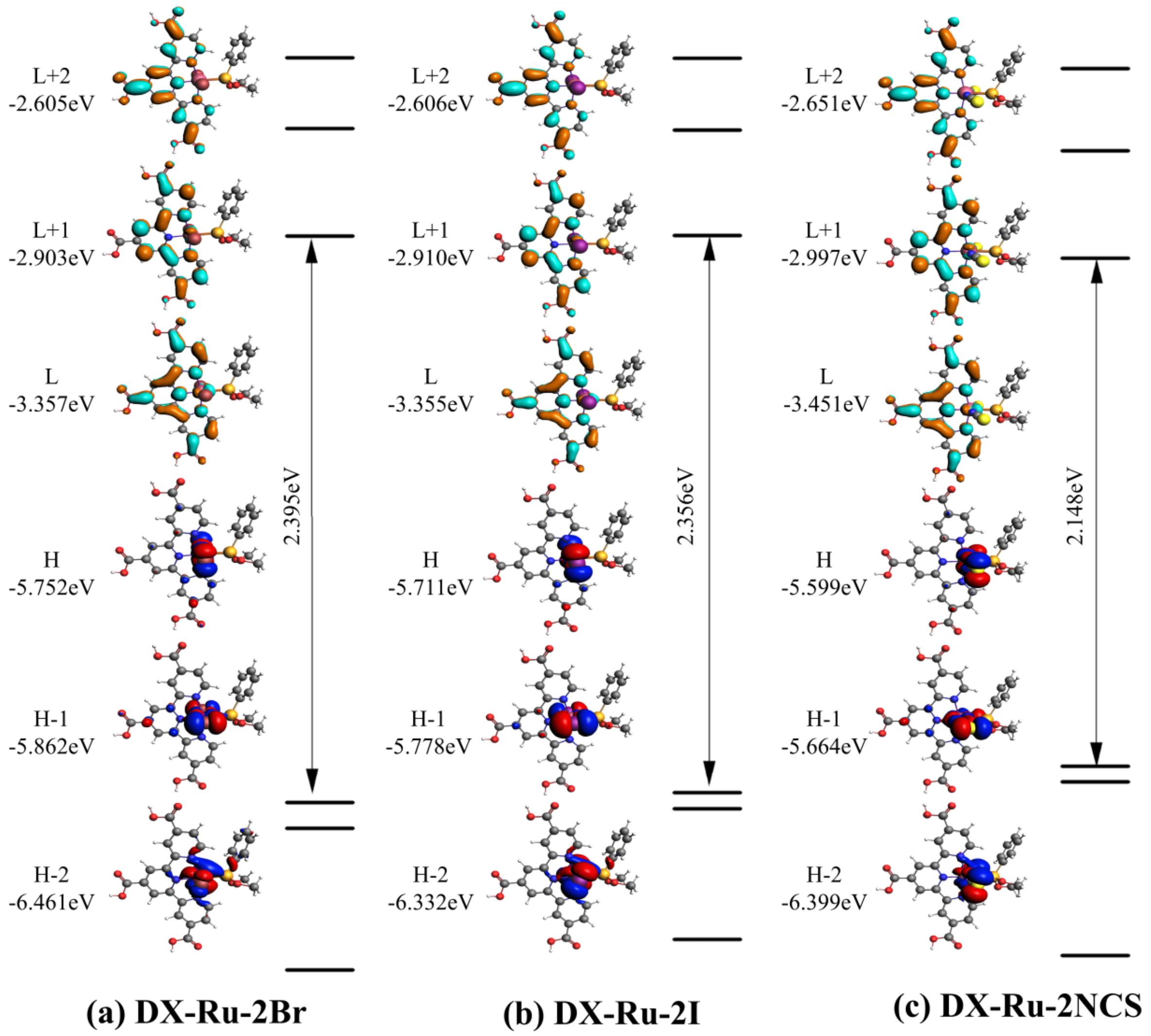

Figure S3. Six frontier molecular orbitals of (a) DX-Ru-2Br, (b) DX-Ru-2I, and (c) DX-Ru-2NCS calculated by SR-TDDFT. Blue, gray, orange, red, white, crimson, purple, yellow and pink spheres represent N, C, P, O, H, Br, I, S and Ru atoms, respectively.

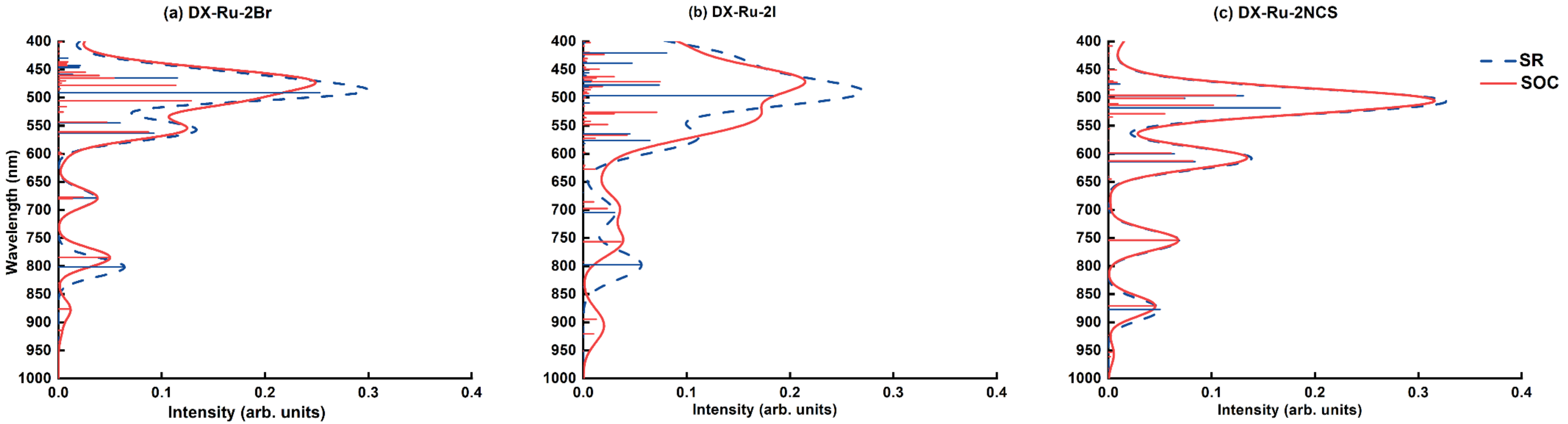

Figure S4. Simulated absorption spectra (λ > 400 nm) of (a) DX-Ru-2Br, (b) DX-Ru-2I, and (c) DX-Ru-2NCS complexes in DMF. Excitation energies and spectra calculated by SR-TDDFT are shown in blue and excitation energies and spectra calculated by SO-TDDFT are shown in red.

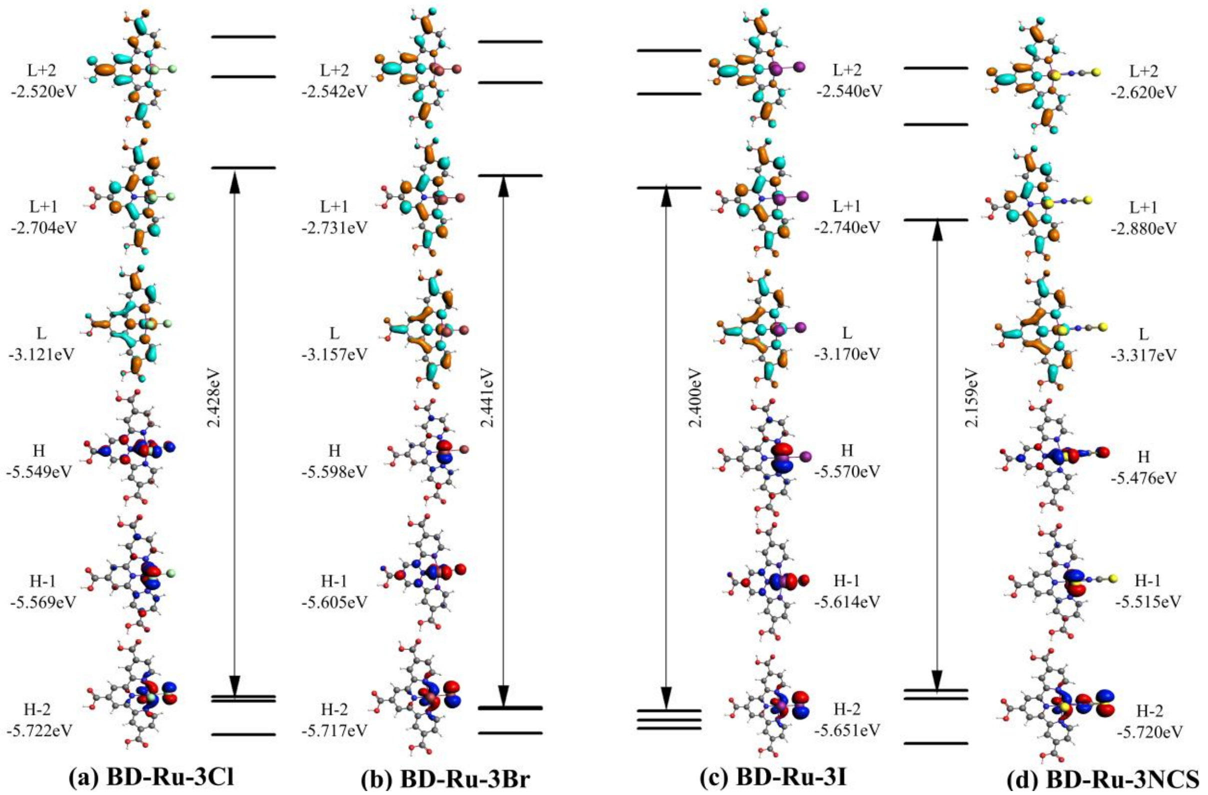

Figure S5. Six frontier molecular orbitals of (a) BD-Ru-3Cl, (b) BD-Ru-3Br, (c) BD-Ru-3I, and (d) BD-Ru-3NCS. Blue, gray, orange, red, white, green, crimson, purple, yellow and pink spheres represent N, C, P, O, H, Cl, Br, I, S and Ru atoms, respectively.

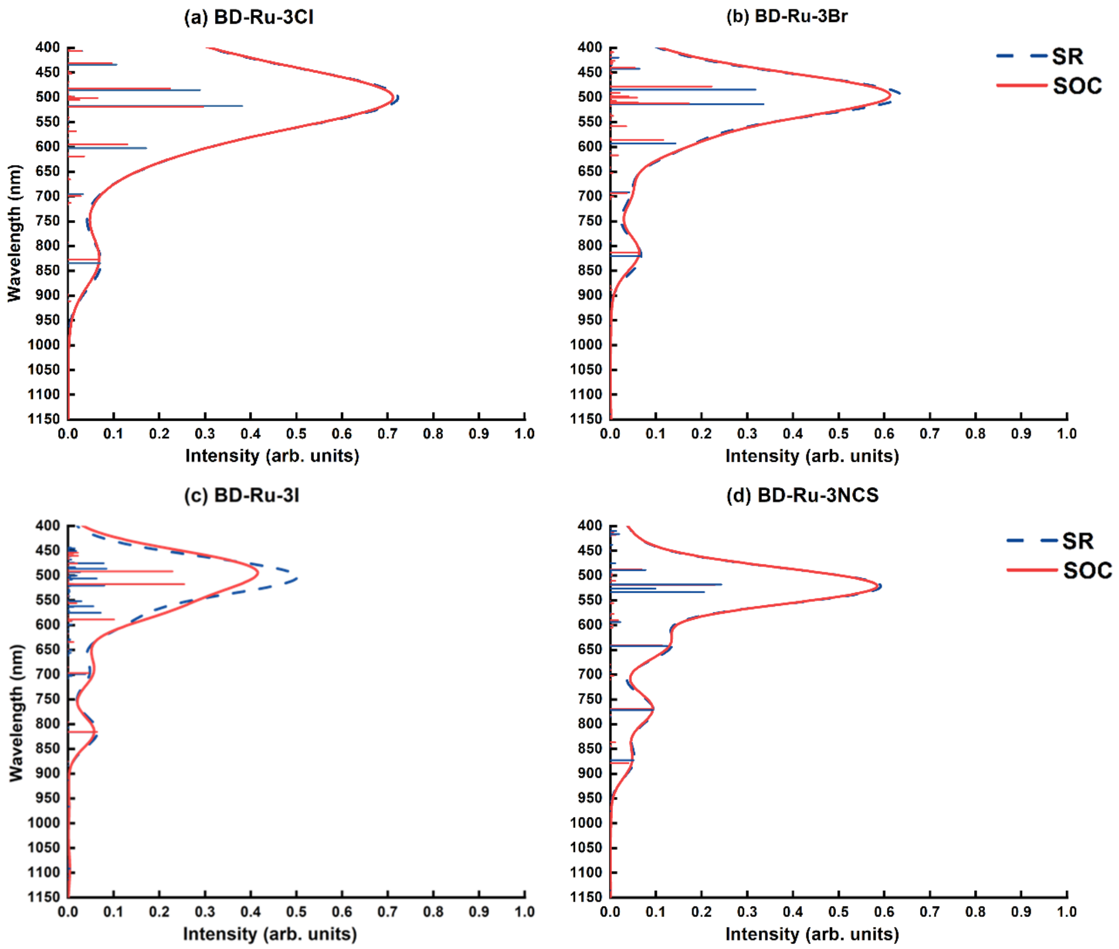

Figure S6. Simulated absorption spectra (λ > 400 nm) of (a) BD-Ru-3Cl, (b) BD-Ru-3Br, (c) BD-Ru-3I, and BD-Ru-3NCS complexes in DMF. Excitation energies and spectra calculated by SR-TDDFT are shown in blue and excitation energies and spectra calculated by SO-TDDFT are shown in red.

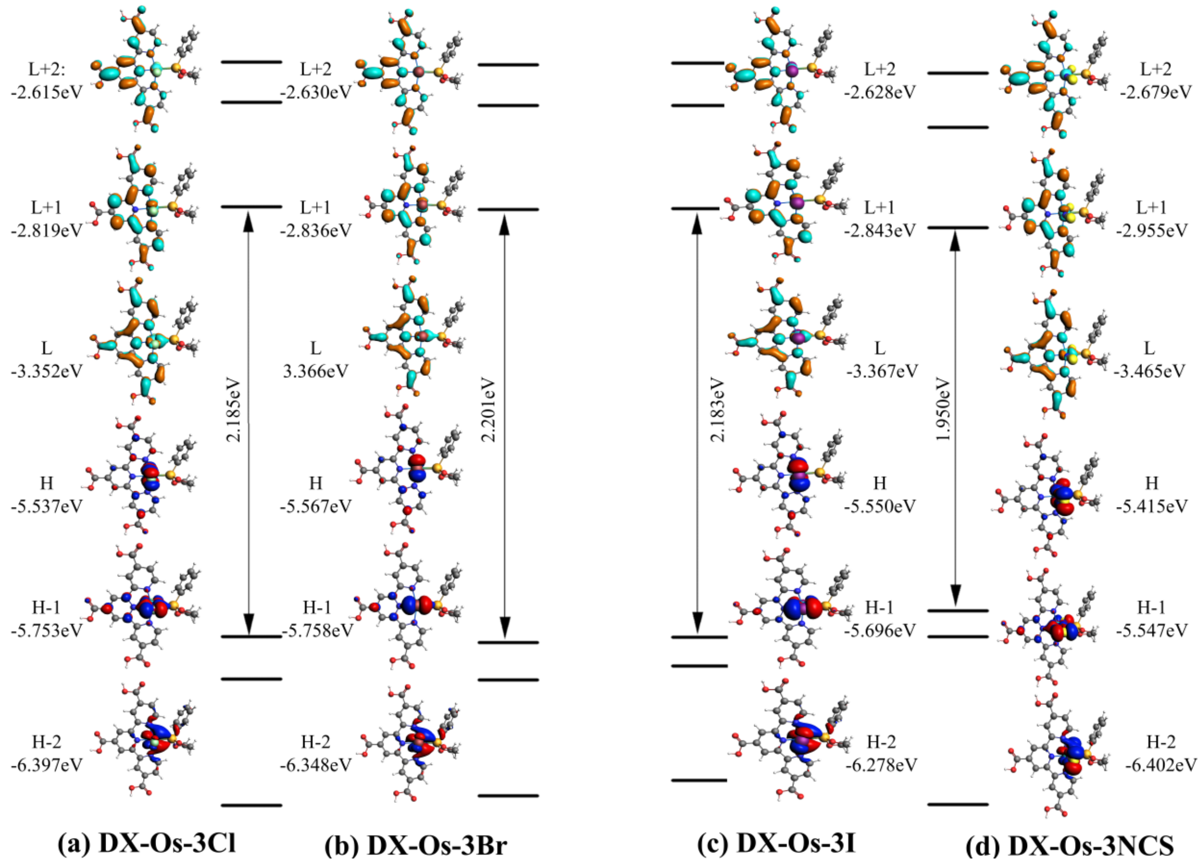

Figure S7. Six frontier molecular orbitals energy level of (a) DX-Os-2Cl, (b) DX-Os-2Br, (c) DX-Os-2I, and (d) DX-Os-2NCS. Blue, gray, orange, red, white, green, crimson, purple, yellow and cyan spheres represent N, C, P, O, H, Cl, Br, I, S and Os atoms, respectively.

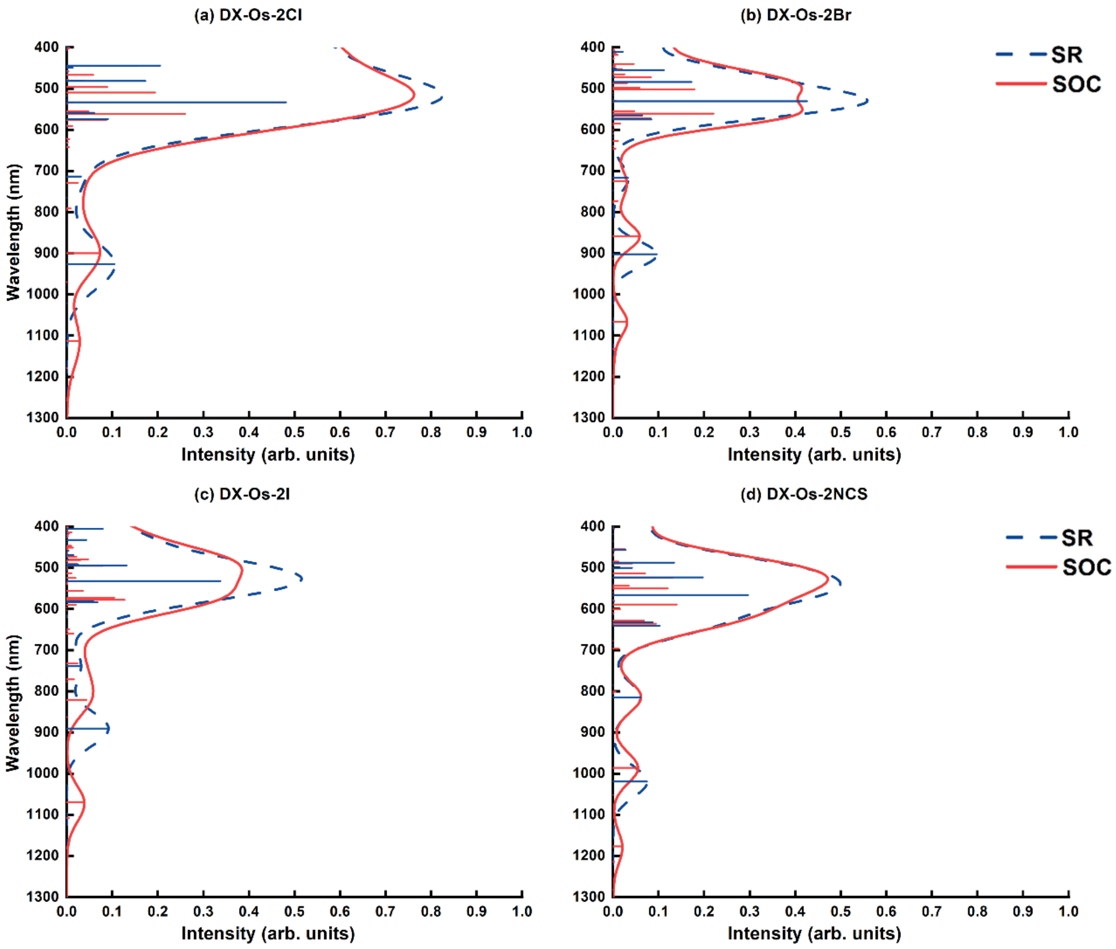

Figure S8. Simulated absorption spectra (λ > 400 nm) of (a) DX-Os-2Cl, (b) DX-Os-2Br, (c) DX-Os-2I, and DX-Os-2NCS complexes in DMF. Excitation energies and spectra calculated by SR-TDDFT are shown in blue and excitation energies and spectra calculated by SO-TDDFT are shown in red.
